# Affiliative and agonistic interactions distinguish sex-biased social groups of greater spear-nosed bats, *Phyllostomus hastatus*

**DOI:** 10.1371/journal.pone.0356790

**Published:** 2026-08-28

**Authors:** Severine B.S.W. Hex, Gerald S. Wilkinson

**Affiliations:** Department of Biology, University of Maryland, College Park, Maryland, United States of America; Indian Institute of Science Education and Research Kolkata, INDIA

## Abstract

The social environment, and the interactions that comprise it, can have a profound influence on reproductive success and survival. Yet not all individuals experience the social environment in the same way. In polygynous species wherein males and females experience different competitive environments, sex may be an important factor in shaping the degree to which the social environment serves as a source of support or stress. We investigated patterns of affiliative and agonistic interactions in the highly polygynous greater spear-nosed bat (*Phylostomus hastatus*), in which sex-biased social niches may be associated with dramatic female-biased survival, to quantify the extent to which these highly sex-biased social environments differed. Agonistic interactions in male-biased bachelor groups were more frequent and longer in duration than those seen in harem groups. Agonistic interactions also differed qualitatively, with bachelor males almost exclusively engaging in dyadic fights in which they deployed highly physical behaviors, such as “wing-locks”, rarely observed between females. By contrast, allogrooming was only observed between females in harem groups, particularly before emergence in the evening and upon return to the roost. Taken together, these data suggest that the patterns of social interaction in harem and bachelor groups differ in their valence and intensity, with individuals living in bachelor groups experiencing a social environment characterized by more intense aggression without the positive interactions seen between females in harem groups. We hypothesize that these behavioral differences contribute to the sex-biased differences in allostatic load and survival observed in this species.

## Introduction

Sociality is predicted to evolve when the benefits of associating with conspecifics outweigh the costs. Animals living in social groups can benefit from increased mating opportunities, predation dilution, and enhanced resource finding and holding potential [1–3], and social bonds can confer immediate and long-term benefits by buffering individuals from environmental and social stressors [4,5]. However, the intrinsic costs of sociality never disappear. Conspecifics can also be sources of competition for mates and resources [2,6–8], and the resultant conflict inherent in these interactions can contribute to elevated stress and greater allostatic load, the cumulative physiological burden on the body, ultimately leading to higher mortality [9,10]. Such consequences are generally the result of chronic, day-to-day exposure to unremarkable but nonetheless negative social stimuli which, over time, culminate into biological consequences [11–13].

Recent frameworks have suggested that these social experiences can trigger physiological intermediate pathways, such as increased inflammation, elevated hypothalamic-pituitary-adrenal (HPA) axis activity, and epigenetic changes as a result of chronic stress [12,14–16], which, in turn, increase allostatic load as individuals adjust their physiology and behavior in response to transient and/or chronic aspects of their environment [17]. Furthermore, individuals may not experience these stressors and costs equally, and the quality and valence of social interactions individuals face can yield physiological consequences [3]. Sociodemographic factors such as social rank [17–20] and early life social adversity [21, 22] have been associated with substantially shortened lifespans and higher allostatic load, and the accumulation of social experiences across the lifespan can result in unequal fitness and survival outcomes as a reflection of an individual’s social phenotype and the social structure in which they exist.

Sex is one of the most fundamental attributes that can shape an individual’s social experiences. While the typical pattern in mammals is for females to live longer than males, there is great variation between species that is likely the result of socioecological influences [23, 24]. Males and females often face dramatically different regimes of competition, particularly in social systems in which one sex experiences greater intra-sexual conflict. For example, in polygynous mammals and in those with high degrees of male-biased sexual size dimorphism, males are predicted to have an elevated allostatic load and higher mortality risk due to the physiological and social costs of attaining and maintaining dominance [20,24–27]. Moreover, in species with sexually segregated modes of living for much of the year, as in many ungulates [28] and bats [29], males and females can experience dramatically different social and competitive environments, with one sex enjoying more positive interactions (i.e., affiliation) while the other faces greater social costs and negative interactions (i.e., agonism) [30, 31].

In order to link behavior with physiological outcomes and understand the role that differences in social experiences play in shaping physiological consequences, we must first understand whether and to what extent sex-biased social environments differ. To this end, int his study we investigated the social behavior a bat species in which males and females occupy distinct and largely separate social niches: the greater spear-nosed bat (*Phyllostomus hastatus*). The greater spear-nosed bat is highly polygynous, and males are larger than females [32]. At dispersal, young females aggregate into stable groups of 7–25 unrelated individuals who are joined by an adult male, forming a harem in which the male monopolizes >85% of paternity and may defend his position for up to 4 years [32–35]. In contrast to many bat species, the social bonds between these unrelated females often persist for life, even across multiple male turnover events, and females engage in a range of cooperative behaviors, including sharing foraging locations, taking turns “babysitting” creches of pups, and guarding one another’s fallen pups from potentially deadly aggression by extra-harem females [35–38]. In contrast, subordinate, non-reproductive males, called “bachelor males”, live separately from harem groups in less stable bachelor groups, and many males appear to never become harem-males or father young [35]. As a consequence, males and females occupy highly sex-biased social environments; most males spend their entire lives in bachelor groups, while harem groups are composed of females accompanied by the few males that manage to become harem males.

Several lines of evidence suggest that this extreme polygyny may contribute to dramatic sex-biased differences in allostatic load and survival. Females can live twice as long as males, as long as 22 years in the wild, while no male has been found with an estimated age over 12 years [16]. Males exhibit faster rates of DNA methylation change [16] and show higher levels of circulating cortisol and testosterone year-round [39]. Intriguingly, the social status of males also appears to yield differences in survival. Socially subordinate bachelor males have higher mortality rates compared to dominant harem males and exhibit 2.8 times faster rates of DNA methylation change [16], despite harem males having higher levels of circulating cortisol year-round [39]. Together, these findings suggest a potential interplay between sex (male vs. female) and social status (harem male, bachelor male), both of which may be mediated by the social environment (harem groups or bachelor groups) which is largely sex-segregated save for the few males who manage to attain harem male status.

**Fig 1:**
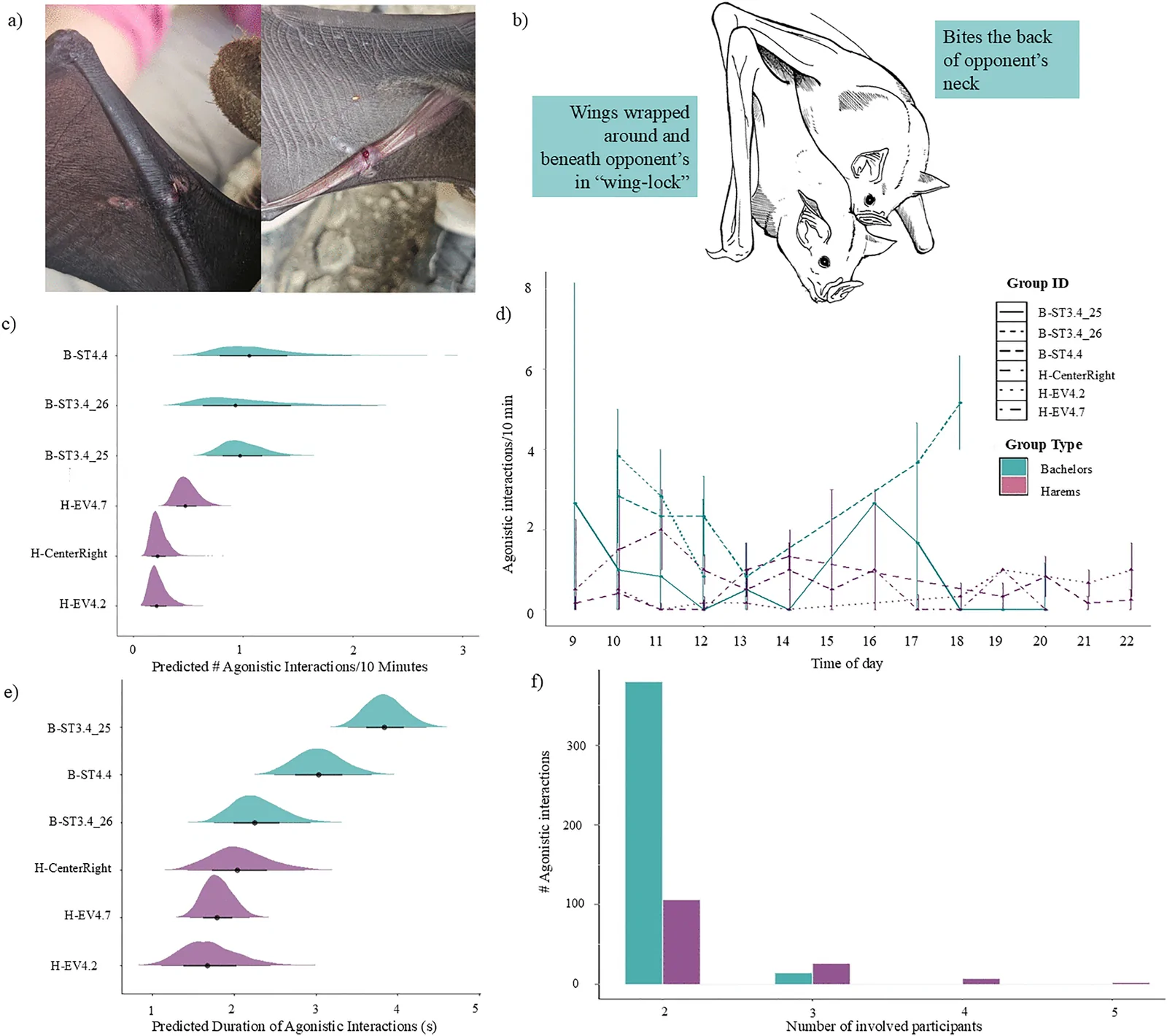
Agonistic interactions in harem and bachelor greater spear-nosed bat groups. a) Examples of injuries found on bachelor males at this location in 2025. Photos taken by S.H. b) Illustration of the “wing-lock”. An opponent is not released from the “wing-lock” until he ceases struggling and falls still, as illustrated. Illustration by S.H. c) Probability density plots showing the posterior distributions of the median rate of agonistic interactions/ 10 minutes. Points represent the estimated medians with the 95% credible interval (CI), and the shaded regions represent the spread of the posterior distribution. The height of the density curve indicates the probability. d) Time course of rate of agonistic interactions. e) Probability density plots showing the posterior distributions of median duration of agonistic interactions, in seconds. f) Histogram of number of individuals involved in agonistic interactions.

What remains understudied are the day-to-day interactions in these two social environments. Therefore, this behavioral study sought to fill this gap in knowledge by characterizing the patterns of affiliative and agonistic interactions in these two distinct types of greater spear-nosed bat social groups, with the goal of illuminating the social environment experienced by individuals in the roost. We have previously observed bachelor males with missing or cracked canines, severe wing wounds including broken wings and punctured wing membranes, and scarring of the face and ears (Fig 1a), which provide indirect clues into the nature of their interactions. This, in conjunction with sex-biased differences in survival and allostatic load, led us to predict higher rates and intensities of agonistic interactions in bachelor groups. In contrast, due to the cooperative and long-term nature of female-female relationships, we expected to observe higher rates of affiliative interactions in harem groups. These findings, while purely observational in nature, provide insight into how differences in sex-biased social environments could culminate into physiological and fitness consequences.

## Methods

### Study animals

Across 11 days in January 2025 and 10 days in 2026 we collected recordings of a wild colony of greater spear-nosed bats living in an abandoned cold storage building in Trinidad, Lesser Antilles (10.5983°N, 61.2117°W). January is the end of the breeding season, confirmed by several observations of female-initiated solicitation and copulation during our study period. Three harem groups (H-EV4.7, H-CenterRight in 2025, and H-EV4.2 in 2026) and two bachelor groups (B-ST3.4 in both 2025 and 2026 and B-ST4.4 in 2026) were selected to serve as focal groups because their roosting locations could be sufficiently illuminated by infrared lights for behavioral coding. At this site, harem groups and bachelor groups roost in spatially separate locations; while all harem groups roost in an abandoned elevator shaft, the bachelor groups roost separately in the adjacent stairwell (Fig S1 in S1 File). We do not have any evidence to suggest that one roosting location is more exposed to the elements or potential predators than the other. In agreement with previous studies, bachelor groups are predominately composed of males at this site. Out of 59 bats captured from bachelor groups between 2024–2026, 5% (N = 3) were young nulliparous females who were in their first year and had not, yet, joined a harem group.

Among the harems, H-EV4.7 had approximately 17 individuals, H-CenterRight had approximately 21 individuals, and H-EV4.2 had approximately 24 individuals. Among the bachelor groups, B-ST4.4 had approximately 32 individuals, and B-ST3.4 had approximately 21 individuals in 2025, and approximately 33 individuals in 2026. As the composition of bachelor groups is unstable, such that group membership is likely to be highly labile across time [38], we treat the groups of bachelors located at ST3.4 to be two independent groups of individuals for analyses. During these same study periods, additional individuals from this population were captured and sampled for other projects, and more information on the focal population is published elsewhere [16].

All animal handling adhered to the methods advised by the American Society of Mammalogists and were approved by the University of Maryland Institutional Animal Care and Use Committee. This research was conducted under a Special Game License given to GSW from the Wildlife Section of the Forestry Division, Ministry of the Environment and Water Resources of Trinidad and Tobago and under University of Maryland (IACUC protocol FR-Jul-24–25#).

### Data collection

Each day we collected two continuous, four-hour video recordings using a Panasonic HC-VX870K 4K Ultra HD Camcorder and a Movo VXR10 universal shotgun microphone paired with a mounted infrared illuminator array. MP4 videos were made at 30 frames per second with a resolution of 1280 x 720 pixels by manually pressing “record” and then leaving the camera on a tripod to record continuously until the battery was depleted. The camera was deployed during two time periods, one in the morning between 9:00–15:00 and a second between 16:00–22:00. Bats were typically out of the roost between 18:00–20:00 for bachelor groups and 19:00–20:00 for harem groups, with the harem male generally last to depart. We obtained a total of 52h of footage from the three harem groups (25h H-EV4.7, 15h H-CenterRight, 12h H-EV4.2) and 62h from the bachelor groups (25.5h B-ST3.4_25, 18.5h B-ST3.4_26, 18h B-ST4.4). 3.5h of footage from H-CenterRight were lost due to a malfunction of the infrared lights (Table S1 in S1 File).

Video was coded for behaviors of interest using ELAN video annotation software (ELAN (Version 6.9) [40] using an all-occurrences sampling scheme to quantify patterns of agonistic interactions and affiliation within harem and bachelor groups. Agonistic interactions were operationalized as two or more individuals engaging in physical contact that included unambiguously aggressive behaviors such as biting, grappling, pinning, or striking with the wings, often accompanied by distinctive, broadband vocalizations (Fig S2 in S1 File and Audio S1). Duration of each agonistic interaction was scored, beginning when first physical contact was made and ending when the parties separated. We also scored how many individuals were involved in the interaction, defined as the number of individuals who received at least one agonistic behavior within the same continuous bout of aggression.

We coded all occurrences of allogrooming as a proxy for affiliation. Allogrooming was operationalized as the initiator making physical contact with another individual and licking or nibbling the receiver’s body. Duration of the allogrooming bout was scored, starting from the moment the initiator made contact with the receiver and ending when the last grooming contact was terminated.

For both agonistic interactions and allogrooming, initiator and receiver sex and status(s) were classified as either harem male, female, bachelor male, or unknown. When an individual had been previously banded, females could be identified by having a band on the left wing, while males have a band on the right. Unbanded males could be easily identified by their visible genitalia and large, sexually dimorphic chest gland, which is visible as a distinctive, bare patch of skin on their throats and chests (Fig S3 in S1 File) [41]. Based upon our capture records, which indicate bachelor groups typically contain 95% males at this time of year, in the absence of other evidence we assumed unbanded individuals in bachelor groups to be males. Unbanded individuals roosting in the harem group were assumed to be females, as young from the previous breeding season (born in April-May) typically disperse prior to this time. Unbanded individuals who approached the roosting group were conservatively called “unknown” unless their sex could be unambiguously determined. Coding was performed primarily by a single observer (S.H), meaning formal inter-rater reliability for sex/status assignment could not be calculated.

### Statistical analyses

Statistical analyses were conducted using R (v. 4.5.1). Rates of agonistic interactions and allogrooming were calculated from all-occurrences and are reported as rate/10 minutes. To investigate differences in agonistic interactions across the focal groups, we fit two Bayesian multilevel models using the “brms” package [42]. To compare rates of aggression across groups while accounting for overdispersion of the data, we fit a zero-inflated negative binomial brms model with number of agonistic interactions as the response variable, group ID as a random effect to allow for partial pooling, which provides more reliable measures of group differences by pulling more extreme measures toward the population mean [43], and number of 10-minute intervals in which individuals were observable per video as an offset to account for differences in sampling effort. To compare duration of aggression across groups, we fit a lognormal brms model with duration of aggression as the response variable, and group ID as a random effect to allow for partial pooling. We calculated marginal medians and group-level contrasts using the “modelbased” package [44]. We report the results of these models as medians with 95% credible intervals (CI). We evaluated whether contrasts between the groups were significant by reporting the region of practical equivalence (ROPE) [45], which corresponds to a region encompassing a “null” hypothesis. Significance is determined by the percentage of the CI that is encompassed within the ROPE. If this percentage is sufficiently low (smaller than 2.5%) the null hypothesis is rejected. The boundaries of our ROPE were set to [−0.10 to 0.10 SD] for analysis. Both models showed convergence and the Rhat values = 1 (see Supplemental Materials for full model outputs and trace plots).

As we did not observe any allogrooming in the bachelor group with which to statistically compare rates or durations between harem and bachelor groups, we fit a Bernoulli brms model with a logit link function to quantify the likelihood of observing allogrooming. The response variable was whether or not allogrooming was observed (0,1), group ID was set as a random effect to allow for partial pooling, and the number of 10-minute intervals in which individuals were observable per video as an offset to account for differences in sampling effort. In addition, we report the rate and duration of allogrooming in the harem groups.

## Results

### Agonistic interactions

We observed 535 agonistic interactions (394 in the bachelor groups and 141 in the harem groups), with no obvious pattern across the course of the day in the frequency of agonistic interactions (Fig 1d). All agonistic interactions observed within the bachelor group appeared to be between bachelor males (Table 1). Within harem groups, most agonistic interactions appeared to be between females of the same harem (N = 101), though females were also seen engaging in agonistic interactions with unknown extra-harem individuals who approached the roosting location (N = 13). Agonistic interactions between females and the harem male were rare, and we observed two contexts in which these female-harem male fights occurred. In two instances, the harem male directed aggression towards a female who had been persistently soliciting copulations from him. In the remaining five, the harem male had approached two females who were engaged in allogrooming, only to be repeatedly attacked by one of the grooming females each time he approached. Aside from these examples, most examples of harem male aggression were directed towards unknown, extra-harem individuals who had landed near the harem roosting spot (Table 1). These fights were always short (range 0.9 - 5.4s) and decisive, with the intruder invariably retreating. However, more than half of such approaches by strangers never escalated into physical aggression, and the trespasser was repelled after the harem male emitted a vocalization (N = 29).

**Table 1:** Summary of the number of agonistic and affiliative interactions observed in harem and the bachelor groups.

Participants	Agonistic interactions	Allogrooming
**Female – female**	101	197
**Female – harem male**	7	0
**Female – Unknown extra-harem individual**	13	0
**Harem male – Unknown extra-harem individual**	20	0
**Bachelor male – bachelor male**	394	0

Qualitatively, agonistic interactions generally followed different patterns of behavior in the bachelor and harem groups. The most common pattern of male agonistic interactions involved one individual attempting to assume a position behind the other from which they would bite the back, head, or nape of their opponent’s neck while wrapping their wings around their opponents’ wings in a “wing-lock” that rendered escape or retaliation apparently difficult or impossible (Fig 1b and S1 Video). This could not be mistaken for copulatory behavior, as males tuck their wings against their bodies during mating and do not bite the female, while the female has her wings free to engage in a conspicuous wing fluttering display throughout solicitation and the entirety of copulation. The wing-lock position behind the opponent appeared to be one from which an individual was most able to win an agonistic encounter. During fights, combatants appeared to grapple for this dominant position, with the individual beneath in a few cases managing to flip around their opponent to achieve the wing-lock, and most fights were terminated once one of the bachelor males managed to successfully subdue his opponent by rendering him functionally immobile. We even observed males initiating defensive aggression at the mere stimulus of another bachelor passing behind them or resting against their back. Fights generally concluded with the loser ceasing to struggle and being released, after which they would move away from the victor and roost elsewhere in the huddle. Less frequently, males would grapple face-to-face, attempting to box, grasp, and bite their opponent, and in these instances the loser usually flew away (S2 Video).

By contrast, most agonistic interactions in harem groups involved a single female directing aggression towards one or multiple others in the form of bites to the head and back whilst the recipient(s) huddled away and offered no retaliation (S3 Video). Rarely did these agonistic interactions constitute “fights” in which both partners exchanged aggressive behaviors. When fights did occur, we generally did not observe the same grappling and wing-locking seen in bachelor males. Rather, females would exchange bites either face to face or from a lateral position (S4 Video). Intriguingly, many of the agonistic interactions with more than two individuals were initially dyadic interactions which were thereafter joined by a third party. In these cases, the third party appeared to direct her aggression indiscriminately between the original combatants, and occasionally to previously uninvolved bystanders.

We only observed the wing-locking behavior once in a harem group (S5 Video). The incident occurred during peak foraging time, and there were only four individuals in the roost: the harem male who hung grooming himself just outside of the depression in which the harem typically roosted, and three females, two who had been previously roosting in body contact, and a third who returned to the roost later and hung on the other side of the roost deposit. The newcomer crawled over to one of the original females already in the roost, and the two exchanged mutual sniffing. But when the newcomer female moved behind the one she had sniffed, nosing her as if she was attempting to initiate allogrooming, the other of the two original females began producing aggressive vocalizations and pounced on the newcomer, putting her in a wing lock. The female in the wing lock did not vocalize or struggle. Interestingly, the moment the attacker began vocalizing, the harem male approached and interposed between the fighting females, which immediately stopped the fight. The two original females moved to one side of the roost, and the one who had been attacked retreated to the other, while the male hung for a moment in the middle, turning his head between the parties, before he approached the female who had been attacked and sniffed her. As he sniffed her, the female performed wing flutters similar to those females use to solicit copulation, but less exaggerated. After a few seconds, the male returned to where he had originally been hanging and resumed grooming. Subsequently, all three females clustered within body contact of one another and began grooming themselves without further incident.

While most agonistic interactions were accompanied by conspicuous, audible vocalizations, in both harem and bachelor groups we observed a few fights in which the participants did not vocalize as they attempted to bite one another, particularly on the legs or wings. As these interactions were often long in duration and always concluded in one of the individuals either flying away or retreating far from their opponent, we concluded they were agonistic in nature and coded them as agonistic.

While rates of agonistic interactions showed inter-group variation, the bachelor groups expressed significantly higher rates of aggression than the harem groups (Table 2, Fig 1c). Though H-EV4.7 showed a higher rate of agonistic interactions than did both of the other harems, the CIs overlapped with the ROPE suggesting that these differences should be interpreted with caution. There was no significant difference between any of the bachelor groups.

**Table 2:** Summary of the rate and duration of agonistic interactions observed in harem and bachelor groups.

Group	ID	Agonistic interactions/10 min (median [95% CI])	Duration agonistic interactions (median [95% CI] s)
Bachelor	B-ST 3.4 (2025)	0.90 [0.65, 1.30]	6.24 [5.47, 7.20]
	B-ST 3.4 (2026)	0.86 [0.40, 2.24]	3.65 [2.85, 4.81]
	B-ST 4.4	0.98 [0.55, 1.91]	4.93 [4.02, 6.05]
Harem	H-CenterRight	0.20 [0.12, 0.37]	3.31 [2.31, 4.72]
	H-EV4.2	0.20 [0.10, 0.42]	2.71 [1.81, 4.04]
	H-EV4.7	0.44 [0.31, 0.65]	2.91 [2.37, 3.63]

Duration of agonistic interactions was generally higher in bachelor groups than in harem groups, though there was substantial inter-group variation (Table 2). Notably, the bachelor group with the lowest median duration of agonistic interactions (B-ST3.4_26) showed modest overlap with the ROPE of all three harem groups, at 9.6%, 6.5%, and 4.6% for harems H-CenterRight, EV4.7, and H-EV4.2, respectively. While none of the three harem groups differed in duration of agonistic interactions, there was a significant difference between the bachelor groups with the highest (B-ST4.4) and lowest (B-ST3.4_26) durations. The longest fight observed in a bachelor group lasted 124s, compared to the longest fight observed in a harem group, which lasted only 21s.

Most agonistic interactions were dyadic in both harems and the bachelor groups (Fig 1f). However, 18.4% of agonistic interactions in harem groups were triadic (N = 26), 5.1% involved four participants (N = 7), and 1.4% (N = 2) involved five participants. In comparison, only 3.5% of agonistic interactions in the bachelor groups were triadic (N = 14), and we observed no agonistic interactions with more than three participants.

### Allogrooming

**Fig 2:**
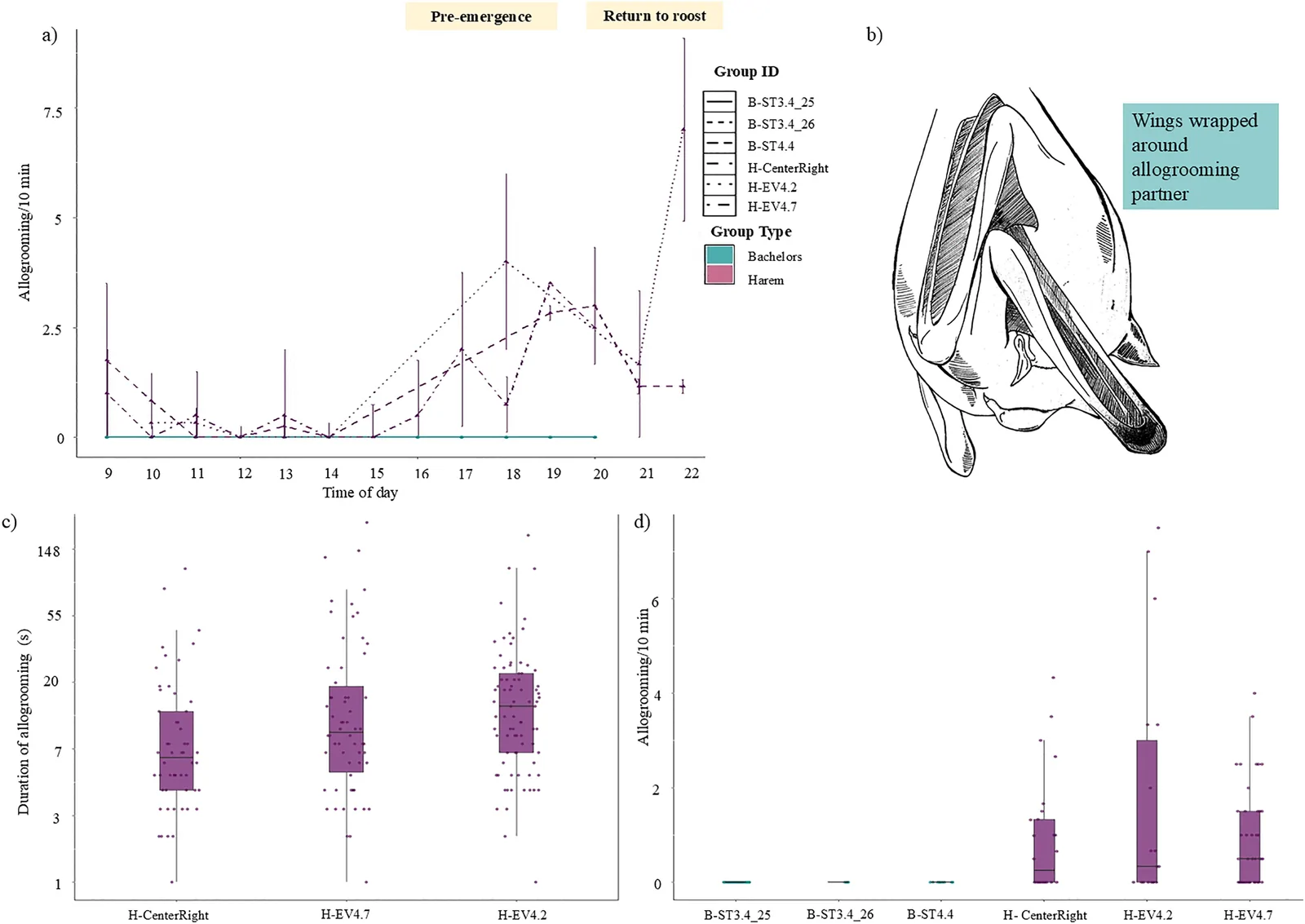
Allogrooming in greater spear-nosed bats. a) Time course of rate of allogrooming. Only harem groups engaged in allogrooming, and showed an increase in rate of allogrooming preceding departure from the roost (16:00-18:00) and upon return (19:00-20:00). b) Illustration of “allogrooming huddle”. c) Duration of allogrooming bouts in the three harem groups. d) Rate of allogrooming. Illustration by S.H.

We observed 197 instances of allogrooming, and only females within harem groups were seen to engage in this behavior. Across all three harems, allogrooming appeared to increase in frequency prior to emergence from the roost for evening foraging (16:00–18:00), and then to remain elevated after return to the roost (19:00–20:00) (Fig 2a).

Allogrooming was most often initiated by one individual approaching the other and stretching forward to sniff at her throat, back of neck, stomach, or anogenital region, occasionally with a conspicuous teeth-baring expression. We observed two distinct types of allogrooming: short alternating exchanges, and longer often mutual “allogrooming huddles” (Fig 2b). Shorter allogrooming bouts were almost always mutual, alternating exchanges in which one individual initiated allogrooming by licking her partner’s neck before tilting back her head for the other to reciprocate, with the dyad often exchanging several rapid alternations (S6 Video). The longer duration “allogrooming huddles” often involved one or both parties enfolding the other in their wings and directing the majority of their grooming to difficult-to-reach locations, such as the back of the wing membranes, the membrane around the feet and tail, the inner wing near the arm pit, the top of the head, and the back (S7 Video). These longer bouts could either be mutual or, less frequently, unidirectional in which the individual receiving grooming also groomed themselves. These allogrooming huddles occasionally involved more than two or even as many as five individuals, and in these instances individuals appeared to not just lick one another but also to bend down and rub their heads along the stomachs of their social partners.

The predicted likelihood of observing allogrooming in any given 10 minute observation window in the three harem groups was 13% [95% CI: 6–25%] for H-CenterRight, 16% [95% CI: 6–34%] for H-EV4.2, and 20% [95% CI: 12–32%] for H-EV4.7, while for the bachelor groups it was nearly 0%, as might be expected given we never observed the behavior in over 60h of video recording. The median duration of an allogrooming bout was 10s (IQR [5–19], range 1-222s) (Fig 2c). Allogrooming within harems occurred at a median rate of 0.5 allogrooming bouts/10 minutes (IQR [0, 1.5], range 0–7.5) (Fig 2d, Table 3).

**Table 3:** Summary of the rate and duration of allogrooming in harem groups. No instances of allogrooming were observed in bachelor groups.

Group ID	allogrooming/10 min (median [IQR])	Duration allogrooming (median [IQR] s)
H-CenterRight	0.25 [1-1.33]	6.5 [4 –13]
H-EV4.2	0.33 [0-3]	9.5 [5.25-19]
H-EV4.7	0.5 [0–1.5]	14 [7 –23]

## Discussion

Day-to-day interactions can profoundly shape whether the social environment serves as a source of social support or stress, and sociodemographic factors such as sex can swing this pendulum by influencing the types of social interactions experienced by individuals. In this study, we sought to examine whether and how affiliative and agonistic interactions differ across social environments in a highly polygynous bat, wherein most males spend their lives in bachelor groups while all females, accompanied by a dominant harem male, live in stable harem groups. As we predicted based on previous work demonstrating sex-biased differences in allostatic load and survival [16,39], we found that the social environments of the two, sex-biased social groups differed markedly in the patterns of social interactions observed among individuals during the breeding season.

Rate of agonistic interactions was significantly higher in the bachelor groups than in the harem groups. In harem groups, most agonistic interactions appeared to be brief, unilateral expressions of aggression toward a passive recipient when a female was attempting to emerge from the roosting cluster or pushing into a preferred position, similar to what has been reported for Asian particolored bats, (*Vespertilio sinensis*) [46]. By contrast, most aggressive interactions in the bachelor groups involved dyadic fights between active participants and were of longer duration, with some lasting two minutes. Rather than engaging in punching or “boxing” with their wing thumbs during agonistic interactions, as is seen in Seba’s short tailed fruit bats (*Carollia perspicillata*) [47,48], greater spear-nosed bats relied more heavily upon their powerful jaws to deliver bites, as has been observed in water bats, (*Myotis daubentonii*) [49]. Individuals in bachelor groups also engaged in physical, full body-contact grapples to subdue and functionally immobilize their opponents in a “wing lock” which, to our knowledge, has not been reported in another bat species. The injuries we have found on bachelor males suggest that participants in these fights can sustain nontrivial damage. Furthermore, almost all agonistic interactions in the bachelor group were dyadic, whereas several individuals were more likely to share the aggression of a single female in harem groups.

Taken together, these findings suggest that the experience of aggression within bachelor groups in this population may be more intense than in harem groups, though future studies are needed to determine if this intensity of aggression is consistent throughout the year. While the current study did not seek to directly link behavior to physiological outcomes, previous studies have suggested that higher allostatic load and mortality is predicted in males when dominance increases fecundity but is costly to attain [14,20,25,26]. Bachelor males achieve little, if any, paternity compared to harem males [32,33], and the intensity of aggression observed within bachelor groups suggests that, if this level of agonism is experienced chronically, there may be high social costs associated with achieving reproductive status, particularly in being able to physically compete with rivals. Body condition is higher in harem males than in bachelor males, and body condition is positively associated with paternity [32], further suggesting that physical competitive ability is advantageous in attaining dominance.

In agreement with previous findings that male bats tend to be less affiliative than females [49–51] we only observed allogrooming between female bats in harem groups. Rates of female allogrooming appeared to be slightly higher than those reported for Jamaican fruit bats *(Artibeus jamaicensis),* Seba’s short-tailed bat, straw-colored fruit bats (*Eidolon helvum*), and Egyptian fruit bats (*Rousettus aegyptiacus*), but not as high as in vampire bats, *Desmodus rotundus* [52], wherein allogrooming appears to reinforce social bonds critical for food-sharing relationships [53]. Most allogrooming bouts were mutual, which has been suggested to require more active coordination than unidirectional allogrooming due to the fact that both parties need to jointly participate in the behavior [54].

Females engaged in allogrooming most often just before departure from the roost in the evening and upon return from foraging later in the night. An explanation for the latter may be that individuals were grooming and eating edible residue, such as pollen, which had become stuck in their harem-mate’s fur during foraging. In substantiation of this hypothesis, on one night we saw individuals returning to the roost visibly covered in pollen, and we observed particularly protracted and frequent allogrooming, including one instance of a single female receiving allogrooming from two partners simultaneously. Allogrooming before departure could serve to reinforce social bonds or facilitate foraging. Indian short-nosed fruit bats (*Cynopterus sphinx*) form “mutual grooming clusters” prior to emergence in the evenings in which individuals coat one another in a mixture of saliva and glandular secretions, which authors suggested may facilitate individual recognition and bond formation within the harem [55]. Similar functions could be at play in female greater spear-nosed bats, who form long-term social bonds with their unrelated harem-mates and are known to forage cooperatively, using harem-specific screech calls to rendezvous with harem-mates at foraging sites [35,36,56]. As rates of self-grooming are also elevated in harem groups prior to departure, the increase in allogrooming could also be, in part, a byproduct of this general elevation in grooming activity.

Allogrooming has been demonstrated not only to reinforce social bonds across mammalian taxa [15,57], including in bats [52,58], but also to reduce heart rate, glucocorticoid levels, and behavioral measures of stress [59–61], thereby potentially buffering individuals from the negative consequences of chronic stress. While the current study is unable to assign causation between participating in affiliative interactions and physiological outcomes, our findings are consistent with the hypothesis that asymmetry in access to allogrooming between social environments and sexes could contribute to the sex-biased differences in allostatic load and survival observed in greater spear-nosed bats.

As a final point, harem males did not appear to engage in disproportionately more agonistic interactions than females within the harem, though this could not be statistically assessed due to our inability to calculate individual rates of agonistic interactions within harems and bachelor groups. In fact, harem males were involved with a small fraction of the agonistic interactions observed within harem groups. By contrast, though harem males roosted in harem groups, we did not observe the harem male to engage in allogrooming. Given our relatively limited observation periods, we cannot conclusively state whether or not harem males receive and/or give allogrooming. In vampire bats, males allogroom infrequently compared to females [50,52]. By contrast, male Honduran white bats (*Ectophylla alba*) [62] and Indian short-nosed fruit bats [55] were found to engage in allogrooming-like behaviors which may serve to mark group-mates for chemical recognition. It is known that male greater spear-nosed bats produce secretions from a sexually dimorphic chest gland which they spread onto females [34,41]. It is possible, then, that harem males do engage in allogrooming or allogrooming-like behaviors at other times of the reproductive cycle, particularly in relation to spreading this secretion onto the females of their harem. Alternatively, even if harem males do not benefit from allogrooming, being exposed to a lower intensity and frequency of aggression compared to that experienced in bachelor groups could contribute to the differences in longevity and body condition observed between bachelor and harem males, an alternative or complementary mechanism warranting future investigation [16].

Our findings suggest that, in this population, the social environment experienced by males in bachelor groups differs from that of females and harem males in harem groups. During our study period, bachelor males experienced a higher intensity of conflict, while only females engaged in allogrooming. We acknowledge that our data are based on a limited number of focal groups that were observed for only a portion of the mating season and, therefore, we cannot assess seasonal variation in social behavior. However, in total the harem groups contained 62 females while the bachelor groups had 86 males, which were collectively observed for over 110 hours. Moreover, capture records of adult males indicate that bachelor males have been found with wounds in at least seven months of the year, though proportionately more wounds have been recorded during the mating season (December and January) than expected given the number of animals captured (pers. obs. G. Wilkinson). We therefore suspect that the rates of interactions we estimated are representative of this time of year. While we are unable to draw causal links between these patterns of social behavior and physiological consequences, our findings, taken alongside the mounting evidence in this species of sex-specific differences in allostatic load [39] and survival [16] suggest that these outcomes may be partially influenced by differences in the social environment and warrant further longitudinal and experimental study. The social environment can influence individual fitness, and by characterizing the day-to-day interactions individuals experience, we can generate testable hypotheses about how those interactions can have physiological consequences and gain an understanding into how patterns of sex-biased mortality evolve across different social systems.

## Supporting information

S1 File Supplemental Materials for “Affiliative and agonistic interactions distinguish sex-biased social groups of greater spear-nosed bats, *Phyllostomus hastatus”.* (PDF)

S1 Video Bachelor-bachelor fight involving a “wing-lock”. The individual who was initially beneath his opponent flips onto the top at 0.06 seconds and puts opponent in wing-lock. The bachelor in the wing-lock struggles but is unable to break free.
(MP4)

S2 Video Bachelor-bachelor fight, with opponents grappling face-to-face, with a few early apparent attempts to grapple into a position from which to wing-lock. (MP4)

S3 Video Agonism in harem group with one female directing aggression towards several non-aggressive recipients. (MP4)

S4 Video Short conflict between two females in a harem group which, at 0.04 seconds, is intervened in by a third party. It thereafter becomes an escalated fight between the third party and one of the original aggressors.
(MP4)

S5 Video Unusual female-female conflict in a harem group involving a wing lock which, at 0.33 seconds, is intervened in by the harem male, who interposes between the aggressing parties. (MP4)

S6 Video Two females (lower left of roosting cluster) engaged in an “allogrooming huddle.”. (MP4)

S7 Video Two females (top right of roosting cluster) engaged in a brief exchange of allogrooming. (MP4)
