# Supplementary material for: Affiliative and agonistic interactions distinguish sex-biased social groups of greater spear-nosed bats, Phyllostomus hastatus
Source: PLoS One. 2026 Aug 28;21(8):e0356790. doi: 10.1371/journal.pone.0356790 (PMC13524302; doi:10.1371/journal.pone.0356790)
Supplement: Supplementary file 1 [file pone.0356790.s001.pdf]

Supplemental Materials for: Affiliative and agonistic interactions distinguish sex-biased social groups of greater spear-nosed bats, *Phyllostomus hastatus*

This PDF file includes:

Figs S1-S10

Tables S1-S12

Legend for Videos S1-S7

Other supporting materials for this manuscript include the following:

Datasets (available on Figshare after publication)

R Script

S1 Fig. Schematic depicting the locations of the focal social groups that were recorded for analysis. Names of the focal groups represent their relative roosting locations in the elevator shaft (EV) and the stairwell (ST), respectively. Groups roost in a consistent location each day, facilitating repeated observations. However, because bachelor group composition may be labile between nights and across years, we treated observations of the bachelor group recorded at ST3.4 in consecutive years as independent samples. Grid cells indicate the locations of reinforcement bars that have become exposed in the concrete ceiling.

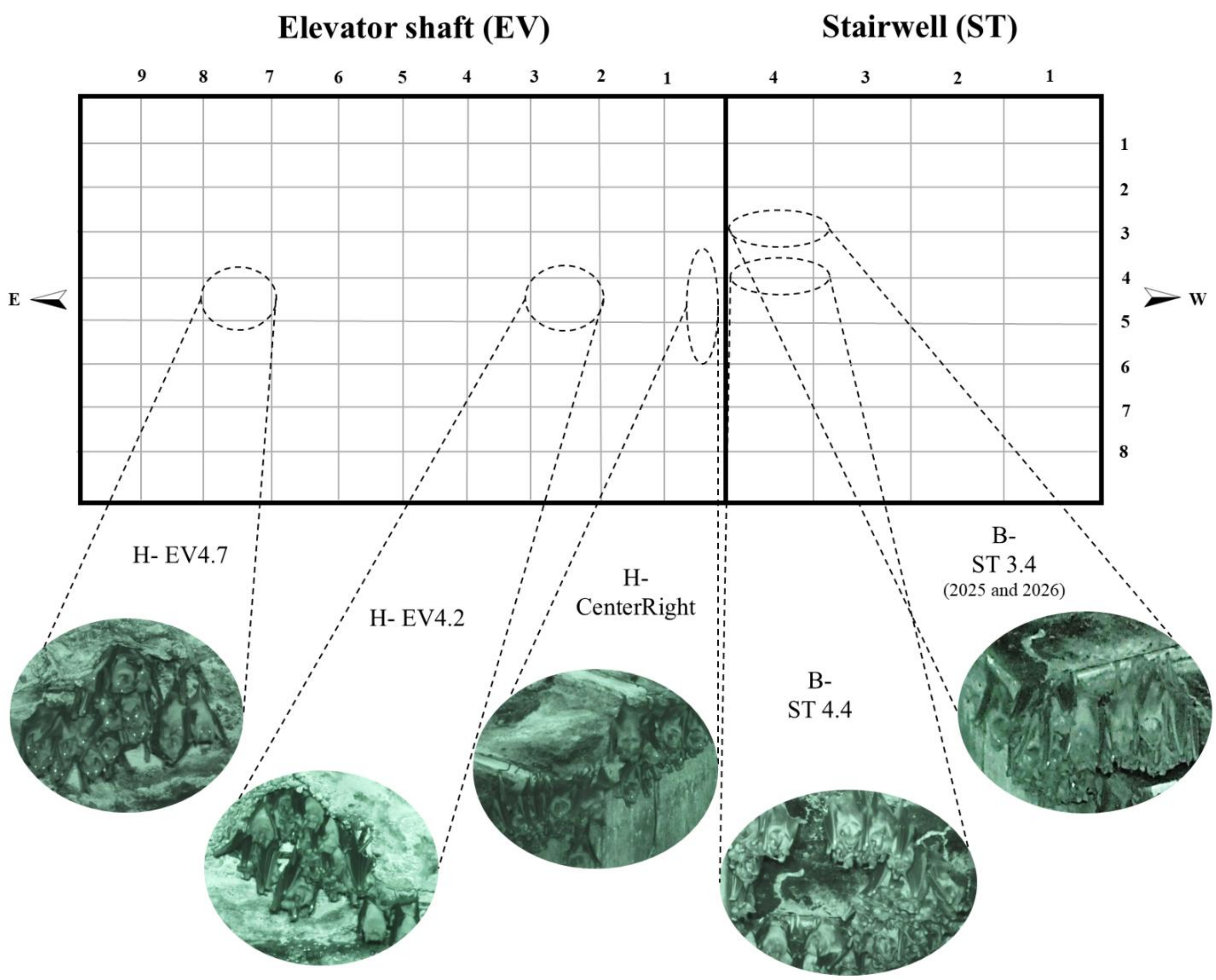

S2 Fig. Spectrogram of vocalizations produced during a bachelor-bachelor fight. Audio, sampled at 48 kHz, was extracted from Video S2, and then a spectrogram was made using Raven Pro 1.6.5 with a Hann window, FFT of 512, and 50 percent overlap.

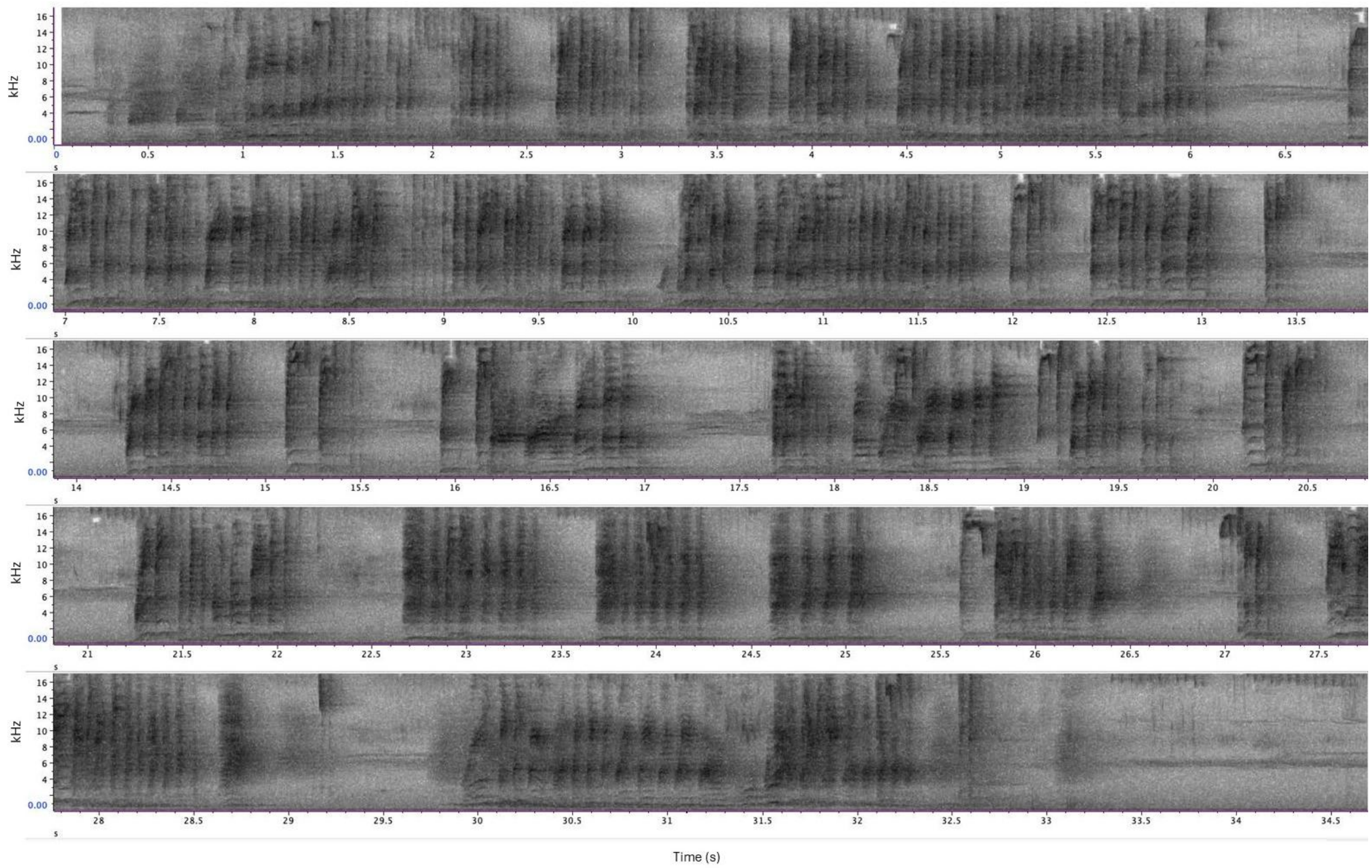

S3 Fig. Example images showing the physical traits used to sex individuals from videos. When an individual's head is lifted to expose the chin and throat, the sexually dimorphic throat gland is clearly visible, appearing as just a dot in females (a) while in males (b) the gland is much larger and surrounded by a conspicuous white patch, which is bare skin. External genitalia are readily observable because the penis is noticeable as a conspicuously bright white dot against the lower body, allowing for reliable sexing if the ventral surface is glimpsed either from the front or side (c, d).

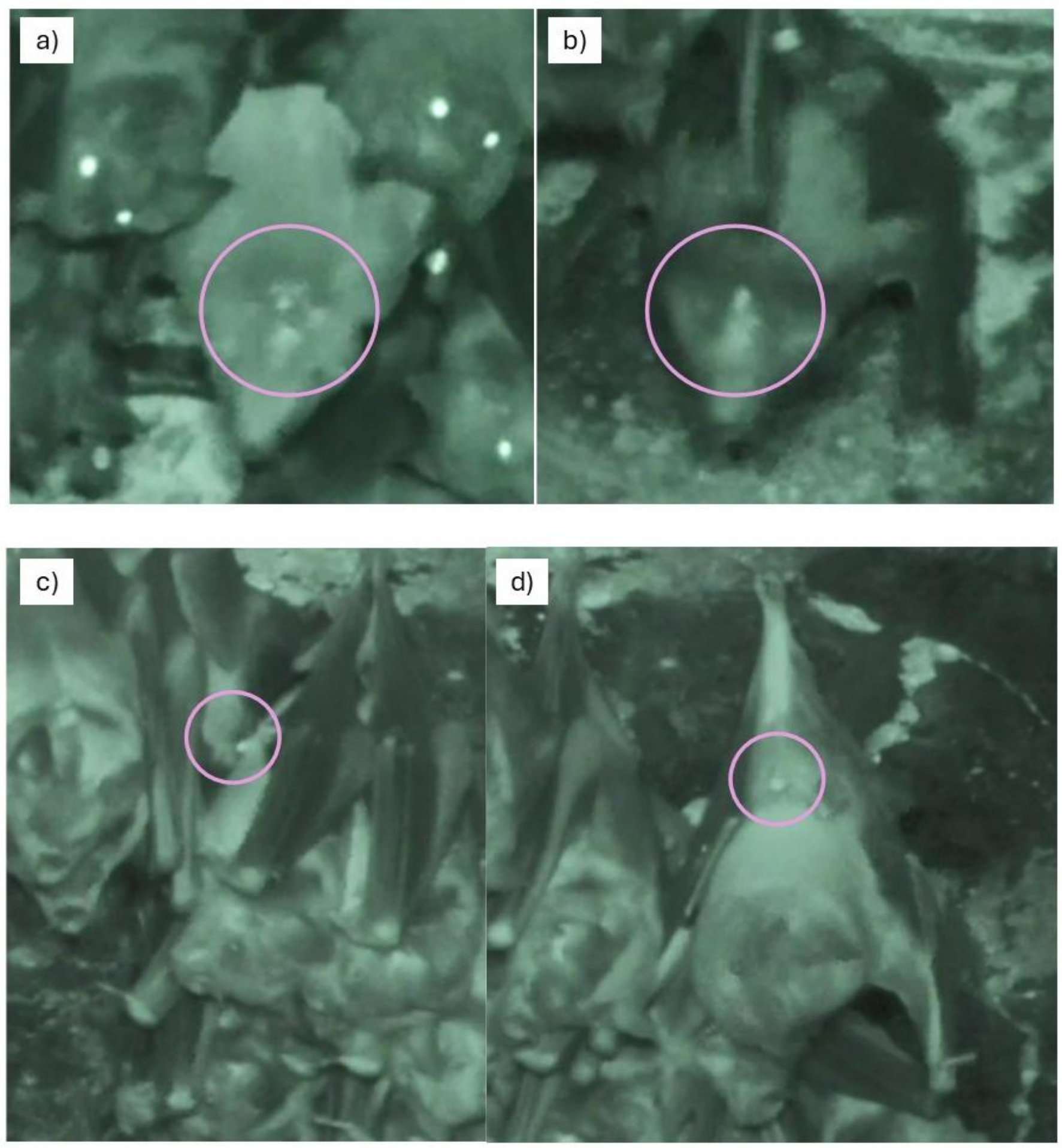

S1 Table. Distribution of number of 30 min videos in which at least one individual was in the roost for harem and bachelor groups in our sample. While we attempted to balance our sampling efforts, due to only coding videos for which there was at least one individual in the roost, we have more PM samples for harem groups than bachelor groups. This is due to bachelor males remaining out of the roost longer than harem members: while the roosts occupied by harem groups were typically empty for about an hour between 19:00-20:00, roosts occupied by bachelor groups were often completely empty beginning at 18:00 and continued to be so even for videos taken after 20:00.

	AM	PM
Bachelor groups	30	31
Harem groups	32	57

Brms Negative zero-inflated binomial model for Frequency of Agnostic interactions/10 minutes

S2 Table. Model Output

Bulk and Tail ESS (effective sample size) measure the sampling efficiencies in the bulk and the tail (95% and 5%) quantiles. Values >1000 are generally considered sufficient for stable estimates. Rhat values of 1 indicate good model convergence.

	Estimate	Est. Error	Lower - 95% CI	Upper - 95% CI	Rhat	Bulk_ESS	Tail_ESS
Intercept	0.31	0.48	-0.69	1.27	1.00	839	1010
shape	0.92	0.22	0.59	1.46	1.00	1969	2254
zi	0.08	0.06	0.00	0.21	1.00	1601	1187

S3 Table. Coefficients

	Estimate	Est. Error	Q2.5	Q97.5
B-ST3.4_25	0.907	0.193	0.531	1.289
B-ST3.4_26	0.886	0.439	0.075	1.809
B-ST4.4	0.989	0.313	0.394	1.636
H-CenterRight	-0.577	0.303	-1.155	0.006
H-EV4.2	-0.609	0.371	-1.320	0.140
H-EV4.7	0.187	0.202	-0.196	0.591

S4 Table. Marginal medians

GroupID	Median	95% CI	pd	ROPE	% in ROPE
B-ST3.4_25	0.90	[0.64, 1.30]	100%	[-0.10, 0.10]	0%
B-ST3.4_26	0.89	[0.40, 2.24]	100%	[-0.10, 0.10]	0%
B-ST4.4	0.98	[0.55, 1.91]	100%	[-0.10, 0.10]	0%
H-CenterRight	0.20	[0.12, 0.37]	100%	[-0.10, 0.10]	0%
H-EV4.2	0.20	[0.10, 0.42]	100%	[-0.10, 0.10]	0.13%
H-EV4.7	0.44	[0.31, 0.65]	100%	[-0.10, 0.10]	0%

S5 Table. Group-level Contrasts

Level 1	Level 2	Median	95% CI	pd	ROPE	% in ROPE
B-ST3.4_26	B-ST3.4_25	-0.03	[-0.65, 1.34]	53.60%	[-0.10, 0.10]	21.16%
B-ST4.4	B-ST3.4_25	0.07	[-0.51, 1.03]	58.4%	[-0.10, 0.10]	25.63%

H-CenterRight	B-ST3.4_25	-0.69	[-1.11, -0.37]	100%	[-0.10, 0.10]	0%
H-EV4.2	B-ST3.4_25	-0.69	[-1.12, -0.34]	99.98%	[-0.10, 0.10]	0%
H-EV4.7	B-ST3.4_25	-0.46	[-0.90, -0.13]	99.58%	[-0.10, 0.10]	0%
B-ST4.4	B-ST3.4_26	0.10	[-1.26, 1.10]	59.33%	[-0.10, 0.10]	18.55%
H-CenterRight	B-ST3.4_26	0.65	[-2.06, -0.15]	99.80%	[-0.10, 0.10]	0%
H-EV4.2	B-ST3.4_26	-0.65	[-2.04, -0.16]	99.65%	[-0.10, 0.10]	0%
H-EV4.7	B-ST3.4_26	-0.42	[-1.79, 0.09]	93.5%	[-0.10, 0.10]	11.76%
H-CenterRight	B-ST4.4	0.76	[1.69, -0.31]	99.98%	[-0.10, 0.10]	0%
H-EV4.2	B-ST4.4	-0.76	[-1.71, -0.26]	99.83%	[-0.10, 0.10]	0%
H-EV4.7	B-ST4.4	-0.53	[-1.47, -0.05]	98.75%	[-0.10, 0.10]	1.76%
H-EV4.2	H-CenterRight	-5.76e-03	[0.19, 0.22]	52.75$	[-0.10, 0.10]	76.42%
H-EV4.7	H-CenterRight	0.23	[0.03, 0.45]	98.42%	[-0.10, 0.10]	7.92%
H-EV4.7	H-EV4.2	0.24	[-0.01, 0.48]	97.15%	[-0.10, 0.10]	10.11%

S4 Fig. Posterior predictive check comparing the fit of the model to our observed data for rate of agonistic interactions. Displayed here are estimates of 1000 draws of the model (magenta) and their fit compared to the observed data (black).

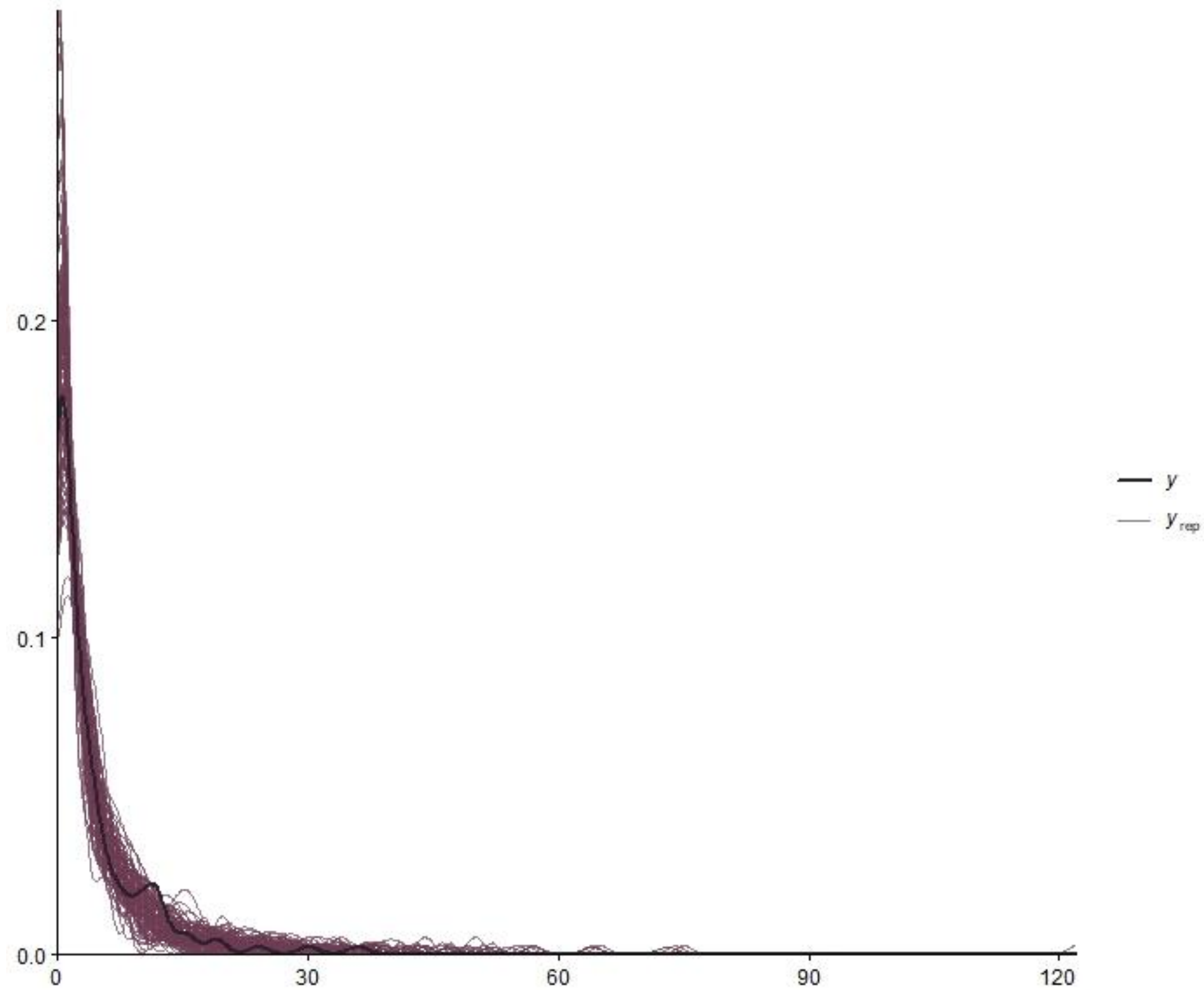

S5 Fig. Traceplots showing convergence for the model evaluating duration of agonistic interactions. X-axis of those trace plots are iterations of the model, and y-axis are all the values of the mean of the posterior distribution assessed by the model.

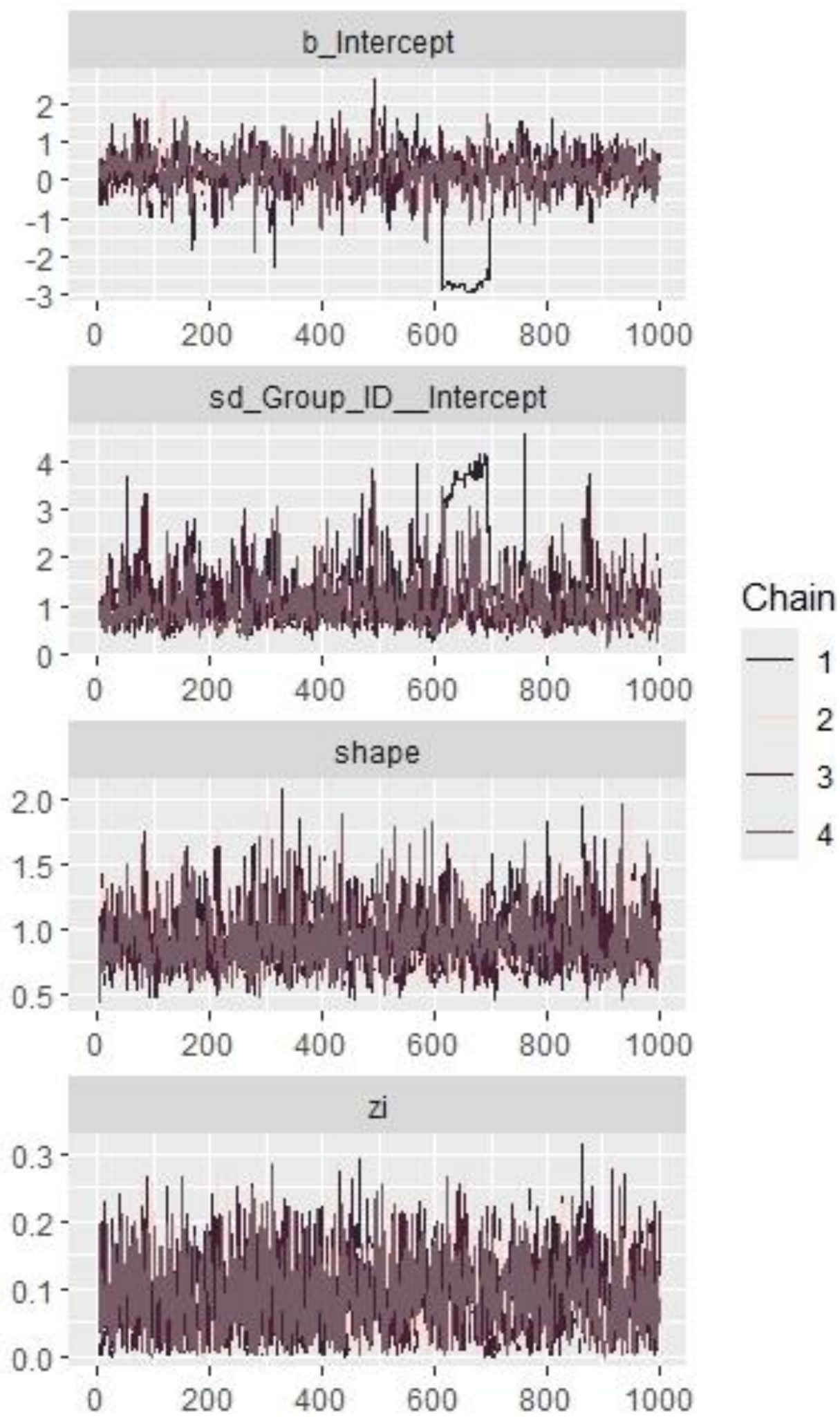

S6 Fig. Density plots showing contrasts in median rate of aggression/10 minutes between the six focal groups. The region highlighted within the dotted lines is the Region of Practical Equivalence (ROPE) [-0.10 – 0.10 SD]. Possessing substantial overlap in the ROPE and 0 suggests that the groups are equivalent, while minimal (< 2.5%) of overlap suggests the groups to be different.

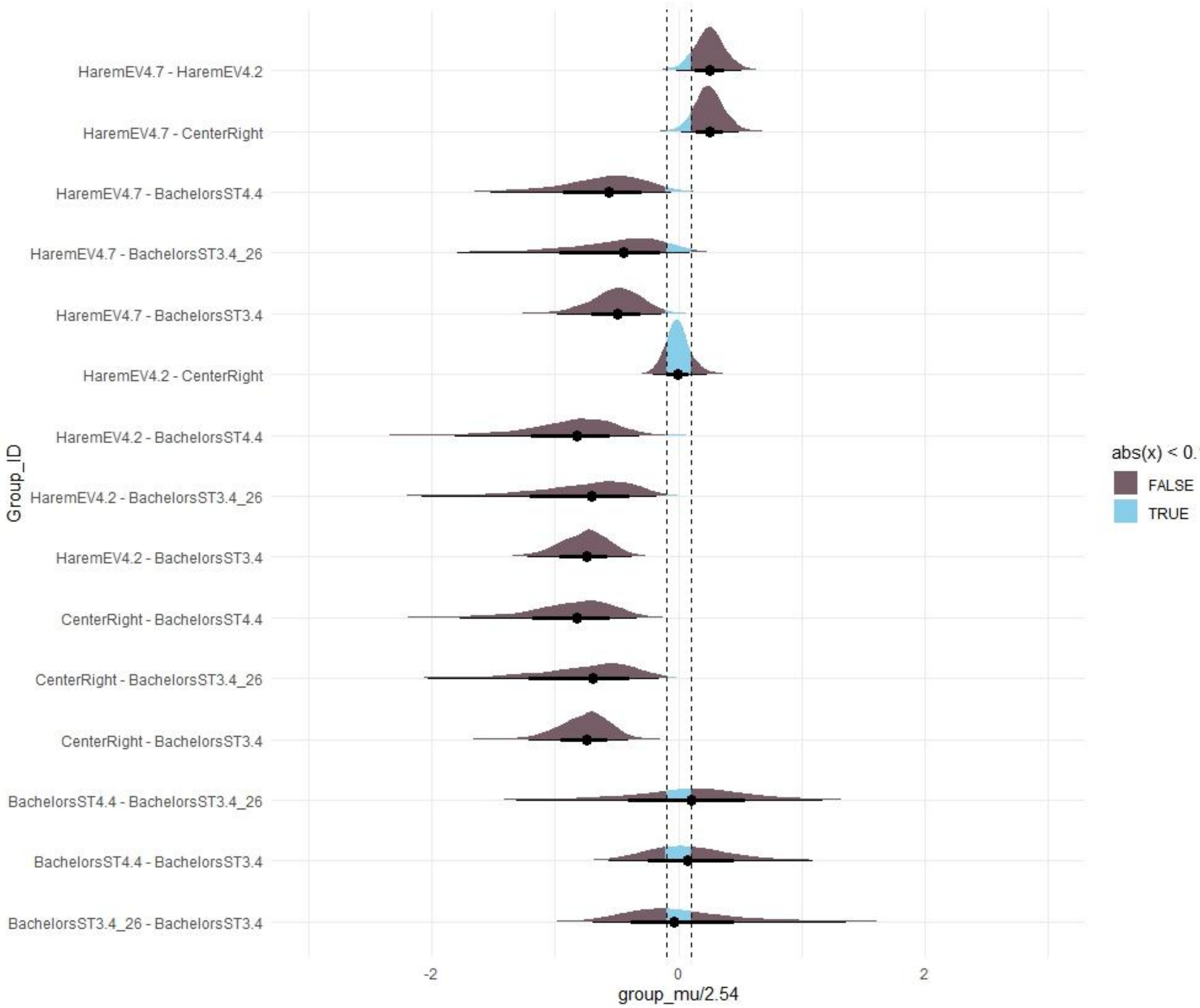

Brms lognormal model for Duration of Agonistic Interactions

S6 Table. Model output

Bulk and Tail ESS (effective sample size) measure the sampling efficiencies in the bulk and the tail (95% and 5%) quantiles. Values >1000 are generally considered sufficient for stable estimates. Rhat values of 1 indicate good model convergence.

	Estimate	Est. Error	Lower - 95% CI	Upper - 95% CI	Rhat	Bulk_ESS	Tail_ESS
Intercept	0.85	0.21	0.42	1.27	1.00	852	783
Sigma	0.99	0.03	0.93	1.05	1.00	2406	2566

S7 Table. Coefficients

	Estimate	Est. Error	Q2.5	Q97.5
H-CenterRight	0.711	0.177	0.357	1.05
H-EV4.2	0.512	0.202	0.106	0.894
H-EV4.7	0.584	0.104	0.377	0.784
B-ST3.4_25	1.345	0.063	1.22	1.471
B-ST3.4_26	0.812	0.132	0.559	1.076
B-ST4.4	1.107	0.100	0.912	1.304

S8 Table. Marginal medians

GroupID	Median	95% CI	pd	ROPE	% in ROPE
H-CenterRight	3.31	[2.31, 4.72]	100%	[-0.10, 0.10]	0%
H-EV4.2	2.71	[1.81, 4.04]	100%	[-0.10, 0.10]	0%
H-EV4.7	2.91	[2.37, 3.63]	100%	[-0.10, 0.10]	0%
B-ST3.4_25	6.24	[5.47, 7.20]	100%	[-0.10, 0.10]	0%
B-ST3.4_26	3.65	[2.85, 4.81]	100%	[-0.10, 0.10]	0%
B-ST4.4	4.93	[4.02, 6.05]	100%	[-0.10, 0.10]	0%

S9 Table. Group-level Contrasts

Level 1	Level 2	Median	95% CI	pd	ROPE	% in ROPE
H-EV4.2	H-CenterRight	-0.60	[-2.21, 0.94]	78.42%	[-0.10, 0.10]	7.63%
H-EV4.7	H-CenterRight	-0.39	[-1.89, 0.77]	73.98%	[-0.10, 0.10]	11.37%

B-ST3.4_25	H-CenterRight	2.92	[1.36, 4.25]	99.95%	[-0.10, 0.10]	0%
B-ST3.4_26	H-CenterRight	0.36	[-1.24, 1.77]	67.50%	[-0.10, 0.10]	9.21%
B-ST4.4	H-CenterRight	1.61	[-0.03, 3.07]	97.38%	[-0.10, 0.10]	0.89%
H-EV4.7	H-EV4.2	0.21	[-1.15, 1.31]	63.65%	[-0.10, 0.10]	12.18%
B-ST3.4_25	H-EV4.2	3.52	[2.07, 4.84]	100%	[-0.10, 0.10]	0%
B-ST3.4_26	H-EV4.2	0.93	[-0.54, 2.39]	90.10%	[-0.10, 0.10]	4.55%
B-ST4.4	H-EV4.2	2.20	[0.66, 3.66]	99.75%	[-0.10, 0.10]	0%
B-ST3.4_25	H-EV4.7	3.31	[2.36, 4.32]	100%	[-0.10, 0.10]	0%
B-ST3.4_26	H-EV4.7	0.74	[-0.32, 1.96]	91.70%	[-0.10, 0.10]	6.50%
B-ST4.4	H-EV4.7	2.02	[0.90, 3.25]	100%	[-0.10, 0.10]	0%
B-3.4_26	B-ST3.4_25	-2.58	[-3.76, -1.26]	100%	[-0.10, 0.10]	0%
B-ST4.4	B-ST3.4_25	-1.31	[-2.53, -0.07]	98.12%	[-0.10, 0.10]	0.34%
B-ST4.4	B-ST3.4_26	1.25	[-0.19, 2.63]	96.25%	[-0.10, 0.10]	2.24%

S7 Fig. Posterior predictive check comparing the fit of the model to our observed data for duration of agonistic interactions. Displayed here are estimates of 1000 draws of the model (magenta) and their fit compared to the observed data (black).

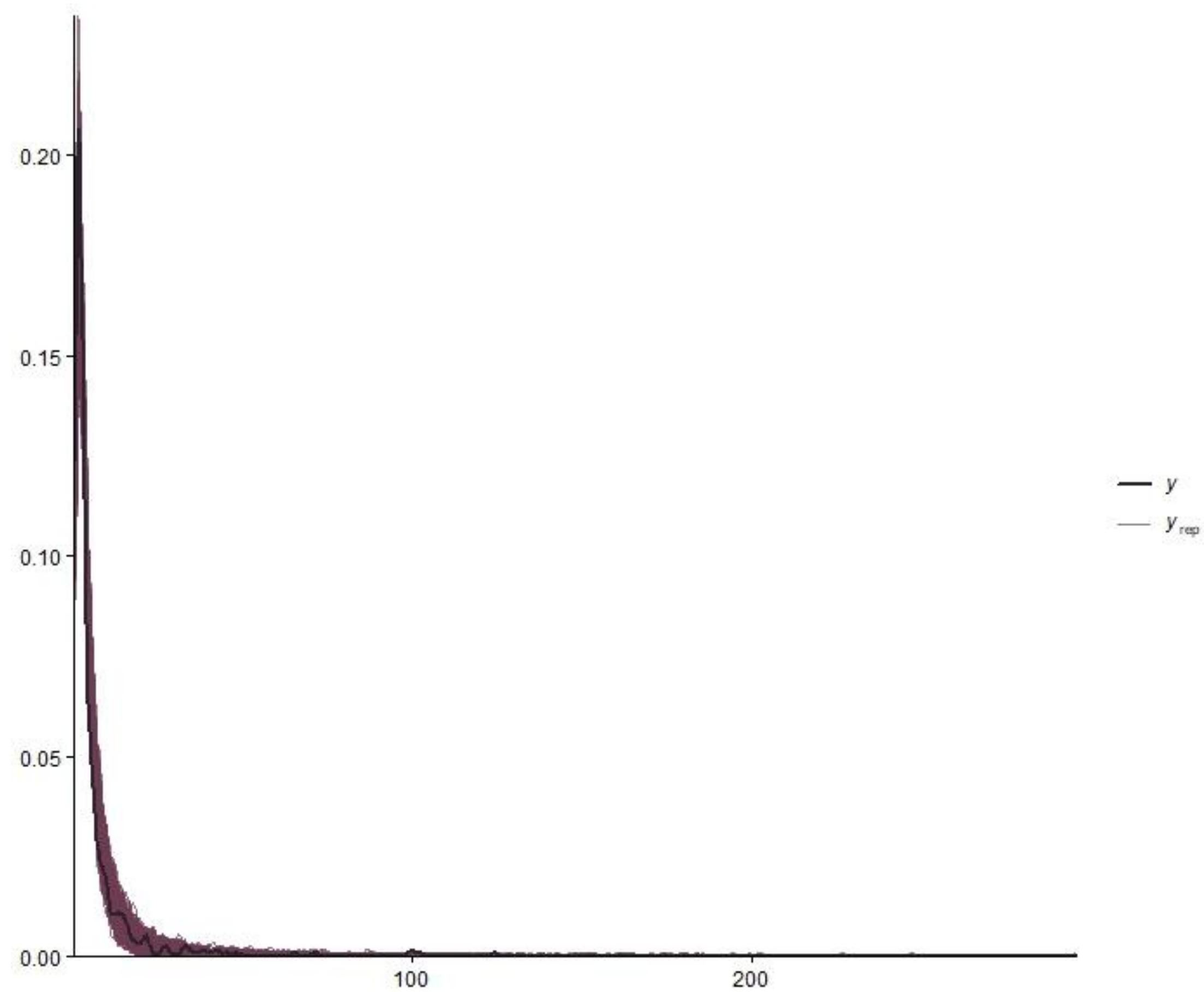

S8 Fig. Traceplots showing convergence for the model evaluating duration of agonistic interactions. X-axis of those trace plots are iterations of the model, and y-axis are all the values of the mean of the posterior distribution assessed by the model.

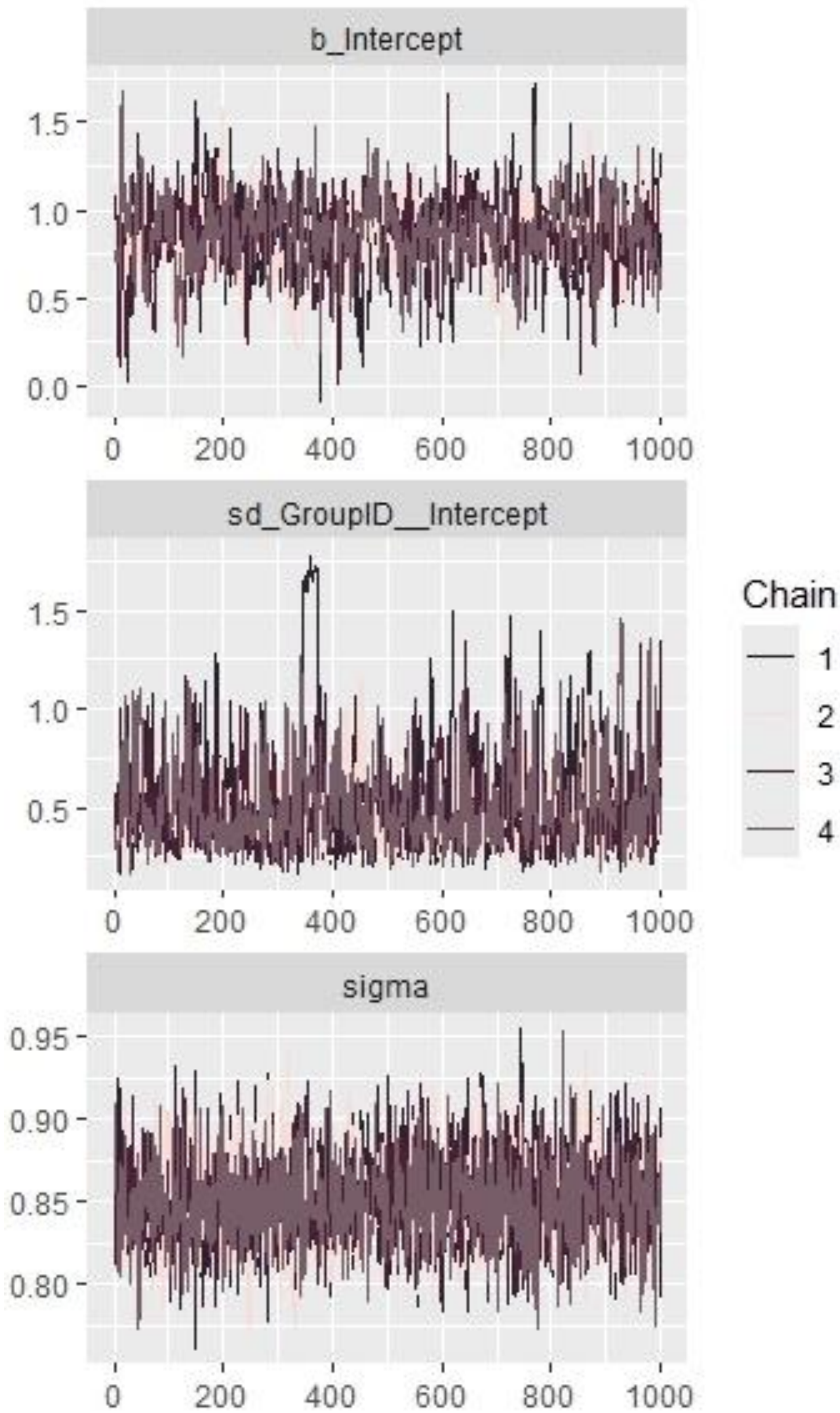

S9 Fig. Density plot showing contrasts in median duration of aggression across the six focal groups. The region highlighted within the dotted lines is the Region of Practical Equivalence (ROPE) [-0.10 – 0.10 SD]. Possessing substantial overlap in the ROPE and 0 suggests that the groups are equivalent, while minimal (< 2.5%) of overlap suggests the groups to be different.

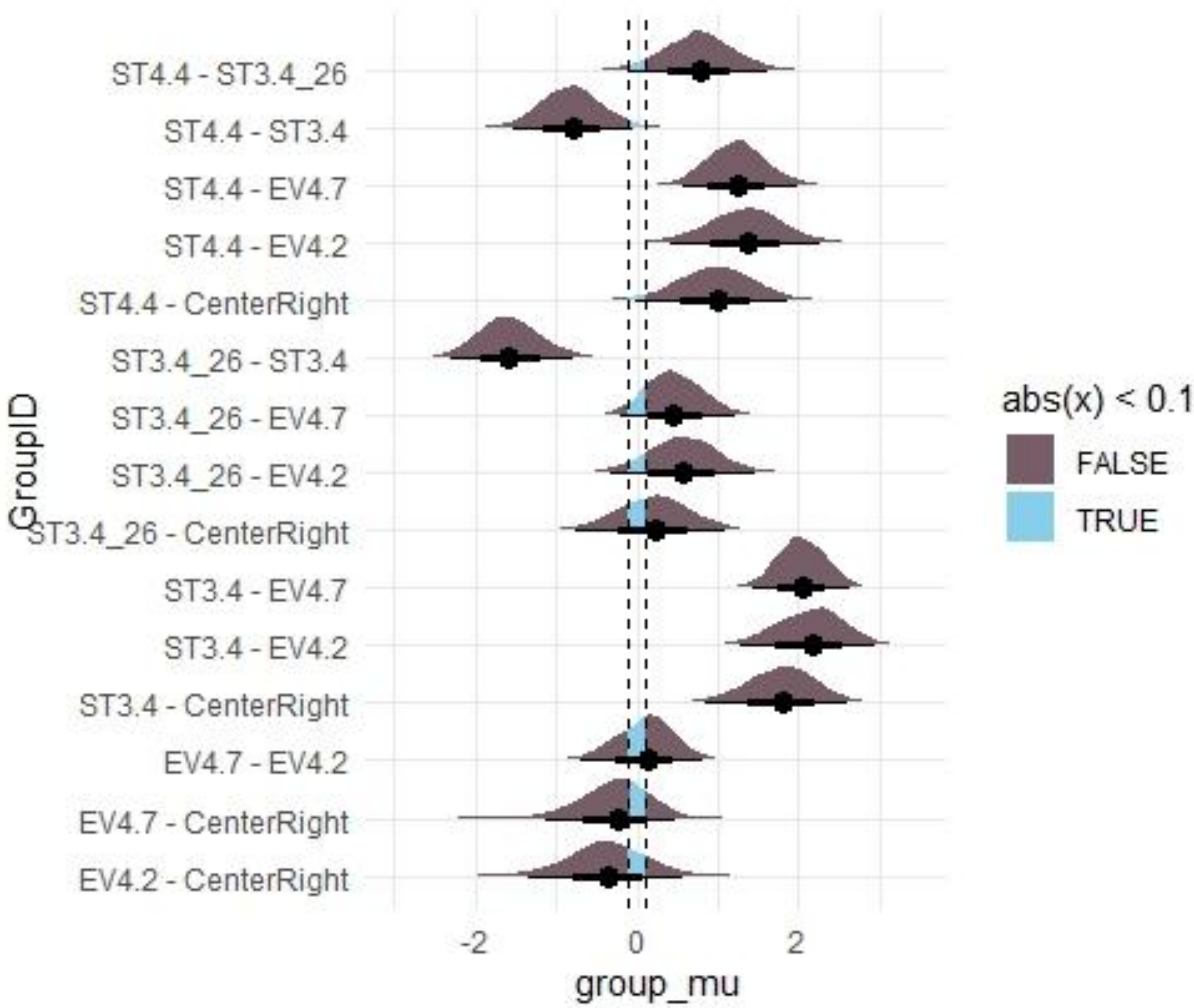

Brms Bernoulli model for probability of observing allogrooming

S10 Table. Model output

Bulk and Tail ESS (effective sample size) measure the sampling efficiencies in the bulk and the tail (95% and 5%) quantiles. Values >1000 are generally considered sufficient for stable estimates. Rhat values of 1 indicate good model convergence.

	Estimate	Est. Error	Lower - 95% CI	Upper - 95% CI	Rhat	Bulk_ESS	Tail_ESS
Intercept	-2.76	1.52	-6.14	0.30	1.01	698	905

S11 Table. Coefficients

	Estimate	Est. Error	Q2.5	Q97.5
H-CenterRight	-1.00	0.404	-1.803	-0.204
H-EV4.2	-0.753	0.51	-1.776	0.242
H-EV4.7	-0.449	0.309	-1.060	0.178
B-ST3.4_25	-7.130	2.814	-14.386	-3.897
B-ST3.4_26	-5.816	3.016	-13.657	-2.030
B-ST4.4	-6.162	2.780	-13.184	-2.716

S12 Table. Marginal medians

GroupID	Median	95% CI	pd	ROPE	% in ROPE
B-ST3.4_25	6.30e-04	[0.00, 0.01]	100%	[-0.10, 0.10]	100%
B-ST3.4_26	2.28e-03	[0.00, 0.05]	100%	[-0.10, 0.10]	100%
B-ST4.4	1.56e-03	[0.00, 0.03]	100%	[-0.10, 0.10]	100%
H-CenterRight	0.13	[0.06, 0.25]	100%	[-0.10, 0.10]	22.79%
H-EV4.2	0.16	[0.06, 0.34]	100%	[-0.10, 0.10]	12.18%
H-EV4.7	0.20	[0.12, 0.32]	100%	[-0.10, 0.10]	0%

S10 Fig. Predicted probability of observing allogrooming in a 10-minute interval across the harem and bachelor groups.

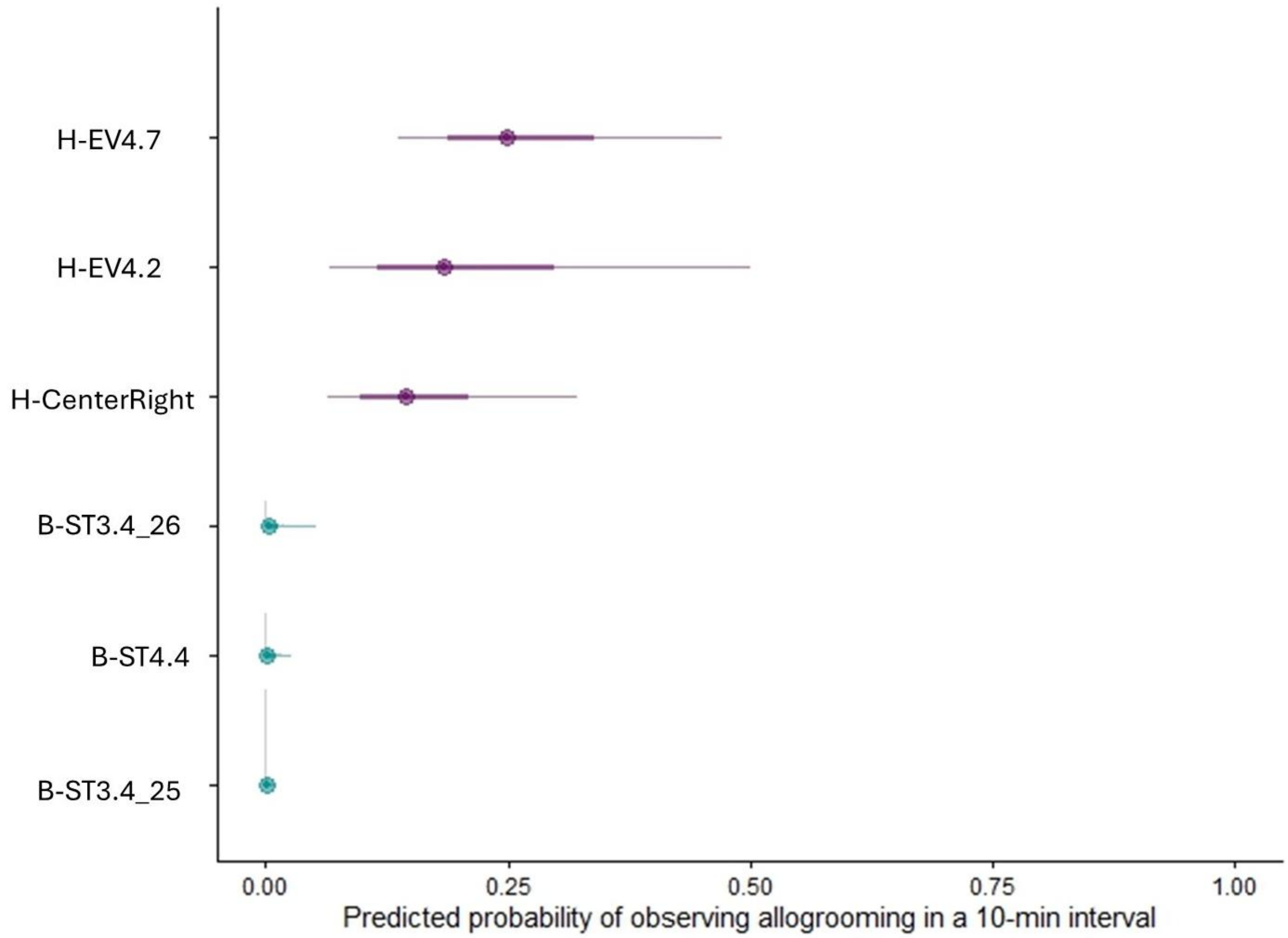

Video legends:

S1 Video. Bachelor-bachelor fight involving a “wing-lock”. The individual who was initially beneath his opponent flips onto the top at 0.06 seconds and puts opponent in wing-lock. The bachelor in the wing-lock struggles but is unable to break free.

S2 Video. Bachelor-bachelor fight, with opponents grappling face-to-face, with a few early apparent attempts to grapple into a position from which to wing-lock.

S3 Video. Agonism in harem group with one female directing aggression towards several non-aggressive recipients.

S4 Video. Short conflict between two females in a harem group which, at 0.04 seconds, is intervened in by a third party. It thereafter becomes an escalated fight between the third party and one of the original aggressors.

S5 Video. Unusual female-female conflict in a harem group involving a wing lock which, at 0.33 seconds, is intervened in by the harem male, who interposes between the aggressing parties.

S6 Video. Two females (lower left of roosting cluster) engaged in an “allogrooming huddle.”

S7 Video. Two females (top right of roosting cluster) engaged in a brief exchange of allogrooming.

Citation:

Hex, Severine; Wilkinson, Gerald (2025). Videos supporting the manuscript "Affiliative and agonistic interactions distinguish sex-specific social groups of greater spear-nosed bats, <i>Phyllostomus hastatus</i>". figshare. Media. https://doi.org/10.6084/m9.figshare.30529634

Other supporting materials: All datasets and R scripts have been deposited in FigShare and are available as of publication.

Dataset S1: All data needed to reproduce the analyses conducted on duration and number of parties involved in aggressive interactions.

Dataset S2: All data needed to reproduce the analyses conducted on rate of aggressive interactions.

Dataset S3: All data needed to reproduce the analyses conducted on duration of allogrooming.

Dataset S4: All data needed to reproduce analyses conducted on rate of allogrooming.

RCode_PhSocialEnvironment: Script used to conduct all analyses

Citations:

Hex, Severine; Wilkinson, Gerald (2025). Datasets needed to replicate all analyses presented in the manuscript "Affiliative and agonistic interactions distinguish sex-specific social groups of greater spear-nosed bats, <i>Phyllostomus hastatus</i>". figshare. Dataset. https://doi.org/10.6084/m9.figshare.30399634

Hex, Severine; Wilkinson, Gerald (2025). R Code for "Affiliative and agonistic interactions distinguish sex-specific social groups of greater spear-nosed bats, <i>Phyllostomus hastatus"</i>". figshare. Software. https://doi.org/10.6084/m9.figshare.30399703
