# Supplementary material for: Affiliative and agonistic interactions distinguish sex-biased social groups of greater spear-nosed bats, Phyllostomus hastatus
Source: PLoS One. 2026 Aug 28;21(8):e0356790. doi: 10.1371/journal.pone.0356790 (PMC13524302; doi:10.1371/journal.pone.0356790)
Supplement: Supplementary file 3 [file pone.0356790.s011.docx]

# Reviewer #1:

 I would like to thank the authors for the opportunity to review this study on the social environment and allostatic load of Phyllostomus hastatus. The integration of high-resolution 4K infrared video with cutting-edge DNA methylation age estimation represents a commendable technical effort to bridge behavioral ethology with physiological aging. The description of male "wing-locking" and the complex "allogrooming huddles" in females provide valuable new insights into the social landscape of this species.
However, the primary concern of this review is a "scaling discrepancy" between the provided data and the broad conclusions regarding chronic survival. Specifically, the study relies on an 11-day behavioral "snapshot" to support claims of lifetime allostatic load and chronic physiological wear-and-tear. Furthermore, the limited group-level replication (using only a single bachelor group) and the application of Bayesian mixed-effects models with a very low number of random effect levels (N=3) raise questions regarding the generalizability and statistical stability of the findings.
While the behavioral observations are rich and technically sound, the manuscript would benefit significantly from a more cautious framing of the causal links between short-term breeding-season aggression and long-term mortality. My detailed comments below are intended to help the authors refine these interpretations and address the statistical limitations inherent in the current study design.
We thank you for your helpful comments and suggestions for strengthening our analyses, as well as for refining our framing to make the scope of our study more clear. We have responded to your suggestions in greater detail below, and all changes to the manuscript are highlighted in green.

Introduction

L35: The authors mention that sociality evolves partly to manage "predation dilution”. In the comparison between harem groups (in elevator shafts) and bachelors (in stairwells), is there a difference in predation risk or environmental stability? If bachelors are more exposed to predators or the elements, this could contribute to mortality independent of agonistic interactions. Please discuss if these ecological factors were considered.

Thank you for this comment. The elevator shaft and stairwell are directly next to one another inside the building, and we do not have any evidence to suggest that one is more exposed to the elements or potential predators than the other. However, whether or not predation or other ecological factors may contribute to differences in mortality was outside of the scope of our current study, which was to characterize differences in the social environment of bachelor and harem groups.

L44-46: The authors state that allostatic load results from "chronic, day-to-day exposure" to social stimuli. However, the study duration is only 11 days (L116). Please clarify how 11 days of observation can be characterized as "chronic" exposure, or revise the text to acknowledge that this study captures a acute peak in social tension (breeding season) rather than a chronic, year-round baseline.

We would like to apologize for having not made this clear in our initial manuscript, and would like to clarify that the intent of our study was not to test hypotheses regarding allostatic load. Rather the concept of allostatic load as a potential consequence of chronic exposure to day-to-day interactions was mentioned to provide background for one reason why it is important to understand the social environment in which animals live and how this may differ between individuals as a consequence of their phenotype. To that end, this study was meant to be a first step toward understanding the types of interactions experienced by individuals in two different social environments, harem and bachelor groups, and whether they differ. Therefore, our snap-shot was not meant to represent chronic exposure, but to provide an initial characterization of differences between social environments in this species to inform future studies. In the text we have endeavored throughout the introduction and discussion to make more explicit that our discussion of allostatic load and social buffering is meant to provide context and potential bases of implications for our behavioral results.

L48-50: Since this study does not measure these physiological markers directly, please ensure the Discussion does not over-interpret "wing-locking" or "grooming" as direct proxies for these complex internal processes without citing the specific limitations of behavioral-only data.

We agree that we cannot use these behaviors as direct proxies, and we have throughout made sure to emphasize the behavioral nature of this paper.

Methodology

L119-121, L125: The study compares two harem groups against only one bachelor group. In behavioral ecology, an N=1 for a social niche makes it difficult to distinguish between a species-wide trait and the unique "culture" or personality dynamics of that specific group. Please justify the inclusion of only one bachelor group and discuss how this limited replication affects the generalizability of the "male-as-stressful" claim.

To address this concern we returned to Trinidad in January, 2026 and then recorded and subsequently scored agonistic and affiliative behaviors in two more bachelor groups and one more harem group, giving us over 110 h of data on 3 harems (containing 62 females) and 3 bachelor groups (with 86 males). We recognize that this is not a large number of groups, but it is comparable to some primate field studies. Increasing the sample size further was not possible at this time because these observations were recorded as a part of a larger study investigating the biological underpinnings of sex-biased aging in bats. As this latter study necessitates capturing individuals and collecting biological samples which disturbs the roosting group, we were limited in the number of undisturbed groups to record. As there are only approximately 20 harem groups and approximately 6 bachelor groups at this site, priority needed to be given to capturing as many bats as possible for these other studies. That being said, after adding observations from more groups, our results remain largely consistent, suggesting that there may indeed be differences in the social environments of harem groups (which are predominately females) and bachelor groups (which are predominately male).

L168-170: The authors assume all unbanded individuals in the bachelor group are male. However, the capture of a female in that exact location indicates a non-negligible presence (~7.7%) of females in supposed male-only spaces. Please provide a sensitivity analysis or clarify the proportion of interactions involving "assumed" males. This is critical for validating the claim that the observed agonistic behaviors are strictly sex-specific.

We agree that we cannot with utmost confidence assume that all individuals of the bachelor groups are males. Previous banding data and published information on this species, however, gives us greater confidence that this is a heavily male-biased space. We have captured groups of bats from the same roosting location (the stairway in the building) on 4 occasions between January 2024 and January 2026. In total, we caught 56 males and 3 females (19 males, 1 female on Jan 6, 2024; 12 males, 1 female on Jan 9, 2025; 7 males, 1 female on Jan 11, 2025; and 18 males, 0 females on Jan 9, 2026). Each of the three females showed no evidence of prior lactation and had unworn teeth, as is typical of animals in their first year prior to joining a harem group, where they remain for the rest of their lives. Consequently, the probability that an unbanded bat observed in a video from that location was a male is 56/59 = 95%.

That being said, we have endeavored to make two changes in the manuscript that we believe strengthen our arguments. First, we have replaced “sex-specific social environments” with the more accurate phrase “sex-biased social environments”, as harems are female-dominated but, by definition, include the dominant male, while bachelor groups are male-dominated but may include a small number of females for short periods, especially following natal dispersal. Second, we have attempted to make it clear that we are attempting to compare the interactions typical of the two different *social environments* rather than *sexes*, per se, though it is important to note from the natural history of this species that these two ways of looking at the question are intertwined. The few females that live in bachelor groups do so only for a short period of time before joining a harem group, while the majority of males live in bachelor groups their entire lives, and even harem males spend several years in a bachelor group. Therefore, while harem and bachelor groups are not necessarily sex-exclusive, membership in each of these group types and the amount of time spent within them is certainly strongly sex-biased and represent fundamental aspects of sex-specific social experiences.

L116- L118: The data collection period is limited to 11 days during the breeding season. The abstract, however, links these interactions to long-term "allostatic load" and "survival" (L30-31). Please temper the discussion to acknowledge that these data represent a seasonal "snapshot." Linking 11 days of breeding-season behavior to lifetime physical wear and tear requires a more cautious interpretation.

As noted above, we added a second year of observation, which spanned 10 additional days of observations. While both sets of observations were conducted during the breeding period, this period should include aggressive interactions given that this is when mating occurs.

Nevertheless, we agree that we are not able to draw direct conclusions about a lifetime of physical wear and tear. We have endeavored to make it clear that this is meant to be a first step in quantifying the behavioral side of the question and thereby motivate future studies capable of identifying causal connections. However, without first knowing whether there were any differences in the social environments experienced by males and females, there would be little justification to further investigate the role of the social environment.

L145-146: There is a significant difference in total footage hours: 40h for harems versus 25.5h for bachelors. Please clarify if the observation windows (AM vs. PM) were perfectly balanced across groups. A lower sampling effort for bachelors increases the risk of "burst bias," where a single morning of high activity could disproportionately skew the results.

Again, as noted above we have added a second year of observation, and three more focal groups (two bachelor groups and one harem group), thereby increasing our total footage hours to 52 h and 62 h for harem groups and bachelor groups, respectively.

That being said, we attempted to balance our data collection efforts across these time periods for harem and bachelor groups, though this did not result in perfectly balanced samples. We have more PM samples for harem groups than for bachelor groups. This is in part due to bachelor males remaining out of the roost longer than harem members. We only coded videos for which there was at least one individual in the roost, and while the harem groups were typically empty for about an hour between 19:00-20:00, roosts occupied by bachelor groups were often completely empty beginning at 18:00 and continued to be so even for videos taken after 20:00. Therefore, members of harem groups were simply present in the roost for more of the observation time in the evening.

Below is the distribution of number of 30 min videos in which at least one individual was in the roost for harem and bachelor groups in our sample.

|  | AM | PM |
| --- | --- | --- |
| Bachelor group | 30 | 31 |
| Harem group | 32 | 57 |

L166-167: Identifying the "sexually dimorphic chest gland" via infrared video in a crowded roost is technically challenging. Please report an inter-rater reliability score (e.g., Cohen’s Kappa) to demonstrate that multiple coders reached the same conclusions regarding the sex and status of unbanded individuals.

All but two videos were coded by a single observer (SH) and those two videos were conducted on a harem group rather than a bachelor group, so conducting inter-rater reliability such as Cohen’s Kappa is not appropriate. However, the sexually dimorphic chest gland is rather easy to identify when the individual raises their head to look down, which occurs frequently. In males, much more of the throat is bare and therefore appears brighter in the infrared-illuminated video. Below is an image taken from a harem group of a female (top left) in which the gland is just a dot, and male (top right) in which the gland is much larger and surrounded by a conspicuous white patch, which is bare skin. Further, external genitalia are readily observable because the penis is noticeable as a conspicuously bright white dot against the lower body, allowing for reliable sexing if the ventral surface is glimpsed either from the front or side (bottom right and left).

Statistical analysis

L180, L185: The authors utilize "group ID" as a random effect (L180, L185). In Bayesian mixed-effects modeling, estimating the variance of a random effect from only three levels is mathematically unstable. Please justify the use of a random effect with only three levels (N=3). Alternatively, consider treating "Group ID" as a fixed effect to provide more stable estimates (McElreath, 2018).

Thank you for this comment. As noted above, we have added data for three more social groups, which increases the number of groups for which we have observations of agonistic behavior to six. This should be sufficient to benefit from partial pooling without compromising model estimate stability.

L188-189” The authors use the Region of Practical Equivalence (ROPE) to determine significance but do not define the specific boundaries used for this "null" region. Please state the specific range used to define the ROPE so the rejection of the null hypothesis (L192) can be independently verified.

Thank you for bringing this to our attention. Our defined ROPE was included in the model outputs in the supplement, but we agree that we should have also explicitly stated it in the manuscript. We have added that our boundaries for the ROPE are -0.10 to 0.10.

L194-195: The manuscript uses "descriptive statistics" for allogrooming because zero instances were observed in bachelors. Please include the bachelor group in a comparative model (e.g., a Bayesian Hurdle model). This would allow for a formal statistical contrast, providing a more rigorous basis for the claim that females benefit from social buffering while males do not.

We appreciate this comment, and recognize that if we were trying to test specifically for social buffering or some other outcome that might be predicted by rate of allogrooming, a comparative model would be preferable. However, as mentioned above, the purpose of this manuscript is simply to describe patterns of social interaction and the differences between harem and bachelor groups. Therefore, we instead ran a brms model with Bernoulli distribution and a logit link function, converting our response variable to whether or not we observed any allogrooming (1,0) within a given observation window to produce a predicted probability of observing allogrooming in each group type.  This analysis yielded the following output, which tells us there is nearly 0% chance of observing allogrooming in bachelors, and a 20-30% probability of observing it in the harem groups in a 10 min period. We have added this result to the supplement and now report the group-level median rates and durations of allogrooming.

L179: The use of a zero-inflated Poisson model (L179) may not account for "overdispersion" if fights occurred in highly concentrated bursts. Please clarify if the data was tested for overdispersion. If the variance of the aggression rate significantly exceeds the mean, a zero-inflated Negative Binomial model may be more appropriate.
Thank you for raising this. We tested for overdispersion of the rate of aggression and as you suggested, the data were overdispersed, so we re-ran the model as a zero-inflated negative binomial. This did not change our findings that all bachelor groups exceeded all harem groups in rate of aggression, nor that there was no meaningful difference within bachelor groups or harem groups.

Results

L250-256: Harem EV4.7 exhibited a rate of aggression more than double that of Harem CenterRight. Given this significant variation within female groups, how can the authors be certain that the higher rate in the bachelor group is a function of sex-specific niches rather than group-specific environmental factors?

While it is possible that group-specific factors, such as individuals of different ages or exposure to water dripping through the ceiling, could contribute to behavioral variation within harem and bachelor groups, adding more observations from another year, which nearly doubled the observation time, resulted in similar patterns as before. In our opinion, the evidence is consistent with sex-biased social environments differing quantitatively and qualitatively in patterns of affiliation and agonism.

L259: The text mentions Harem 'EV3.4.' Is this a typographical error intended to be Harem EV4.7 (as mentioned in L124 and L250), or was a third harem group included in the duration analysis?

This was a typographical error, thank you for pointing this out. This has been changed.

L277-280: Authors describe 'allogrooming huddles' that involve grooming difficult-to-reach locations. Do you have data to suggest these huddles occur more frequently after agonistic interactions? This would provide direct evidence for the 'social buffering' hypothesis.

This is an interesting idea, and while we have not formally tested whether this appears to be the case, it would certainly be interesting to do in a future paper intended to directly test the social buffering hypothesis. Anecdotally, it does not appear as though allogrooming is obviously more likely to occur after an agonistic interaction, though this lack of an obvious conciliatory function does not necessarily provide direct evidence against the potential for social buffering.

L265-268: Authors note that female groups have more multi-party fights (up to 5 individuals) compared to the strictly dyadic fights of males. Does this suggest that female social stress, while less physically 'intense,' is more socially 'widespread' across the group?

This is certainly possible, and raises the interesting question of whether being briefly involved in receiving aggression distributed among many or directly receiving all of it is more or less stressful would be an interesting focus for future research. On one hand, it certainly could be argued that more females are receiving a stressful stimulus at once, and therefore would suffer the consequences. On the other, it is possible that the more intense and focused aggression is qualitatively different in its physiological effects. The difference between these would likely be difficult to test in wild populations.

Discussion

L336-338: The authors provide a compelling observation regarding a spike in grooming when individuals returned covered in pollen. Please discuss whether allogrooming in this species is primarily a functional/hygienic response to foraging residues. If the behavior is largely driven by "cleaning edible residue," it may be premature to characterize it as a "social factor contributing to differences in allostatic load" (L357) without further evidence of its psychological impact.

We would like to clarify, and have done so in the text, that allogrooming in fact shows the greatest elevation prior to emergence, and this level of allogrooming remains elevated after return, which is when we do observe pollen consumption. Based upon our current evidence and the prevalence of allogrooming throughout the day, especially before emergence, we are inclined to interpret most allogrooming as social in nature, with consuming edible residue on fur as a secondary and specific context in which allogrooming may occur.

L354, L381: Throughout the Discussion, behavioral observations are linked to physiological wear and tear (allostatic load). While the authors acknowledge that they cannot assign causation, the concluding sentences suggest a definitive link between "day-to-day interactions" and "physiological outcomes" (L386). Please temper the conclusion to reflect that while the correlations are consistent with the allostatic load hypothesis, this study provides no direct physiological data to confirm the mechanism in this specific population.

Thank you, and we hope that we have made clear that our study is not meant to test the allostatic load or social buffering hypotheses but merely to provide the first behavioral evidence that there are differences in social environment, which could potentially have consequences for male and female physiological outcomes. We have attempted to make clear, when we do make broader links to allostatic load and other physiological outcomes, that these are still hypotheses based upon previous research and pending further investigation.

L356-358: The manuscript suggests that allogrooming is a key factor in buffering allostatic load and promoting survival. However, the authors also note that harem males do not participate in allogrooming (L362) yet maintain superior body condition and survival compared to bachelors (L319-320). Please address this discrepancy. If harem males achieve high survival without allogrooming, does this imply that the absence of aggression is a more significant determinant of allostatic load than the presence of affiliative buffering?

Thank you for bringing this interesting question up. At present, we do not have data to determine which of these underlying causal mechanisms is associated with the body condition and survival of harem males. However, we would like to point out that the longest-lived harem males still live only half as long as females, and so there are clearly many differences which are likely due to a combination of physiological and experiential factors that contribute to observed differences in survival, allostatic load, and immunity in males and females, including between harem males and females. This is why we suggest that allogrooming might be an important social good which, in as much as our data to date suggest, only females benefit from.

L379: The authors cite personal observations to suggest that bachelor wounds occur year-round, justifying the 11-day "snapshot." To strengthen this claim, please provide more detail on these observations (e.g., frequency or seasonality) or cite any available data that suggests male aggression levels do not significantly drop outside the breeding season.

To address this question we scanned our capture records which include nearly 1,000 adult males captured between 1990 and 2026 in eight different months from Dec-July. We noted a total of 50 instances of conspicuous bite wounds on bats, with examples in every month except March, when we had few captures. The highest incidence of bats with wounds occurred in December (30%, N = 6) and Jan (7%, N = 364). We have added this information to the text (Lines 462-471)

# Reviewer #2:

Dear authors, I very much appreciate studies on social behaviour conducted in the wild. Phyllostomus hastatus is a fascinating species with respect to sociality. Although the observation period is relatively short and the sample size limited, I think your study provides valuable observations that can serve as a starting point for further research on the relationship between social environment and allostatic load (including physiological consequences and ultimately fitness). Below I provide several minor comments.
Thank you for your positive comments. We agree with the reviewer that this study is limited primarily in the short study window, though sample size has been improved slightly by the inclusion of two more bachelor groups and one more harem group collected in 2026. All changes in the manuscript directly pertaining to your comments are highlighted in blue.

Abstract
“Valence” is only mentioned in the abstract. I recommend either removing the term or introducing it earlier in the manuscript (e.g., in the Introduction).
Thank you for the suggestion. We have added mention in the introduction.

Methods
Line 116: “In” should be lower case.

Thank you, this has been revised.

Line 141: Please provide the sampling rate and bit resolution of the recordings. Were recordings automatically triggered? If so, based on which threshold?

Thank you for this question. We have added the following information to the manuscript.

MP4 videos were made at 30 frames per second with a resolution of 1280 x 720 pixels by manually pressing “record” and then leaving the camera on a tripod to record continuously until the battery was depleted.

Figure S2:
Please include the spectrogram settings used (e.g., window type, FFT size, overlap). Also specify which software was used to generate the spectrograms.

Thank you for this comment. We have added the following text to the manuscript address it:
An audio file, sampled at 48 kHz, was extracted from a video, and then a spectrogram was made using Raven Pro 1.6.5 with a Hann window, FFT of 512, and 50 percent overlap.

Results
I have a few more general questions regarding the results:
Females appear to solicit copulations. Is this consistently the case in this species? Are there other bat species in which females actively solicit copulations? I am more familiar with species in which males either attempt forced copulations or perform elaborate courtship displays, with females making the mating decision rather than actively soliciting copulation.

We agree that this was an unexpected observation. While we can only speak for this point in the mating season, every instance of copulation we observed was female initiated in a stereotyped manner, with the female approaching and attempting to maneuver herself underneath the male while fluttering her wings and arching back her head and neck in a rhythmic motion. This fluttering solicitation could last for more than a minute before the male mounted, and would continue throughout copulation. We are only aware of one other species documented to have female initiated copulation (Greater short-nosed fruit bat *Cynopterus sphinx*), but they do not appear to have as exaggerated a ritualized solicitation. We are planning to submit a short note describing this behavior in greater depth.

Were you able to determine whether females had preferred grooming partners?

Thank you for your interest in this question. Unfortunately, we do not have the ability to reliably identify individuals from the videos in order to test this question.

I found the fighting behaviour between males particularly interesting. The wing-lock position seems difficult for the attacked individual to counter. Are similar fighting behaviours known from other bat species?
At present, we have not encountered descriptions of a similar grappling and “wing-lock” position in other species, though we did not conduct a systematic literature review of descriptions of male-male agonistic behavior. That being said it is quite possible that the behavior remains undescribed due to the difficulty of studying social behavior of bats in the wild and the relative sparseness of behavioral data available for the immense diversity (~1500) of bat species.

Discussion
Line 319:
Could you briefly clarify how body condition was defined?

While we did not include estimates of body condition in this study, in other studies we have used residuals from sex specific least squares regression of body mass on forearm length.

Line 377:
I would suggest rephrasing this statement — the observation period most likely does not fully capture seasonal variation in social behaviour.

We agree, and have rephrased this sentence per the reviewer’s suggestion (Lines 458-461).
