# Supplementary material for: Affiliative and agonistic interactions distinguish sex-biased social groups of greater spear-nosed bats, Phyllostomus hastatus
Source: PLoS One. 2026 Aug 28;21(8):e0356790. doi: 10.1371/journal.pone.0356790 (PMC13524302; doi:10.1371/journal.pone.0356790)
Supplement: Supplementary file 5 [file pone.0356790.s012.docx]

I thank the authors for their careful and constructive revision. The revised manuscript is substantially improved. In particular, the addition of a second observation period and additional harem and bachelor-group observations directly addresses my earlier concern about limited group-level replication. The revised dataset now provides a more convincing basis for comparing affiliative and agonistic interactions across sex-biased social environments. I also appreciate the authors’ revision of the aggression-rate model to account for overdispersion, their addition of a model for allogrooming occurrence, and their more cautious framing of the behavioural nature of the study.

Overall, I find the manuscript much stronger and closer to publication. The behavioural observations remain valuable, especially the detailed descriptions of male “wing-locking” and female allogrooming. However, I recommend minor revision before acceptance. My remaining concerns are not about the central behavioural pattern, but about ensuring that the interpretation remains appropriately cautious and that several inconsistencies introduced or retained during revision are corrected.

Thank you for your encouraging comments. Below, please find our responses to your remaining suggested changes, including correcting errors introduced during revision.

Recommendation: Minor revision.

The authors have clarified that the study does not directly test allostatic load, social buffering, or survival mechanisms. This is a substantial improvement. However, the Abstract and final Discussion still give considerable prominence to the possibility that the observed behavioural differences contribute to allostatic load and survival differences. In the Abstract, lines 34–39, the sentence linking the behavioural differences to allostatic load and survival should be tempered slightly further. I recommend phrasing this as a hypothesis generated by the present behavioural data, or as a pattern consistent with possible future tests, rather than implying that the present study demonstrates such links. Similarly, in the Discussion, lines 448–455, the authors appropriately acknowledge that causal links cannot be drawn, but the final sentence still implies that the present study characterizes how day-to-day interactions culminate into physiological outcomes. Please revise this to state that the behavioural findings generate testable hypotheses about possible physiological consequences.

We have implemented the suggested wording changes to make more apparent that this paper was meant to be just one more piece in the incremental accumulation of evidence for how and why males and females differ in allostatic load and longevity in this species (Lines 33-36, Lines 458-459)

In the Methods, lines 139–141, the manuscript states that harem groups roost in the elevator shaft and bachelor groups roost in the adjacent stairwell. In the response letter, the authors further explain that they have no evidence that one location is more exposed to predators or environmental conditions than the other. This clarification should be added briefly to the manuscript, either in the Methods or Discussion, so that readers can evaluate whether roost-location differences may confound the harem–bachelor comparison.

Thank you for this suggestion, and we have added a brief clarification in lines 140-141 indicating that we have no evidence about differences in predation risk or other environmental factors.

The expanded dataset is a major improvement. However, in the Methods, lines 136–150, one bachelor roost location, B-ST3.4, was sampled in both 2025 and 2026 and treated as independent because bachelor-group membership is presumed to be labile. This may be reasonable, but it should remain explicit wherever the manuscript refers to “three bachelor groups.” The conclusions should be framed as evidence from sex-biased social environments at this study site rather than as broad species-wide generalization.

We have implemented the above suggestions, and throughout the results and conclusion removed reference to “three bachelor groups”. We have also clarified in our discussion that these patterns are only directly supported by evidence from this population (Lines 378-380, 442).

The shift from “sex-specific” to “sex-biased” social environments is appropriate. The capture data also support the claim that bachelor groups are strongly male-biased. However, in the Methods, lines 185–195, some sex assignments still rely on assumptions from roost membership and visual criteria. Please state clearly that coding was performed primarily by a single observer and that formal inter-rater reliability for sex/status assignment could not be calculated. If possible, the representative image showing the chest-gland/genitalia criteria should be included in the Supplementary Information.

We have added this information both to the main text (Lines 196-197) and have added the images showing chest glands and genitalia in the Supplemental Information as Fig S3.

In the Methods, lines 159–168, the increased total footage reduces the earlier concern about burst bias. However, because AM/PM sampling was not perfectly balanced, I recommend including the AM/PM distribution table from the response letter in the Methods or Supplement. This would make the sampling structure transparent to readers and would help show that the higher aggression rate in bachelor groups is unlikely to be an artefact of a single active time period.

Thank you for the suggestion, and we have added this table to the Supplemental materials as S1 Table.

In the Methods, lines 217–223, and Results, lines 338–344, the added Bernoulli model is an acceptable improvement for a descriptive study. However, the manuscript should make clear that this model estimates the probability of observing allogrooming in a 10-minute interval. It does not directly test social buffering. Any wording linking allogrooming to buffering or allostatic load should remain explicitly hypothetical.

We have made sure that the text and supporting material make explicit this test estimates the probability of observing allogrooming (Line 339).

In the Discussion, lines 416–436, the response to the harem-male discrepancy is only partly satisfactory. If harem males have better condition/survival than bachelor males but were not observed to participate in allogrooming, this suggests that reduced exposure to intense bachelor aggression may be an important alternative or complementary mechanism to allogrooming-based buffering. I recommend that the Discussion state this more clearly. Allogrooming should be framed as a potential female-specific social benefit, not as a general explanation for all harem-associated survival advantages.

Thank you for the suggestion. We agree that this is an important alternative hypothesis to the potential physiological benefits of living in harem groups for harem males, and we have added information about both the apparent sex-specificity of allogrooming as well as the lower exposure to aggression (Lines 427-428, 438-441).

In the Discussion, lines 437–448, the added capture-record information on wounds across months strengthens the argument that bachelor males may experience aggression beyond the immediate observation window. However, wound records are indirect evidence and do not quantify year-round aggression rates. The current cautious framing is mostly appropriate, but please avoid implying that equivalent aggression rates persist outside the mating season.

We were careful not to imply that we think aggression rates are the same throughout the year by explicitly stating that we believe our results are representative of this time of the year and reporting that instances of wounding seems to be higher during the mating season, from which our current study samples. We have attempted to use wording that does not allow further interpretation beyond what we have explicitly stated (Lines 447-453).

Several statistical and terminology issues should be corrected. In the Methods, lines 197–200, the text still refers to “three focal groups,” although the revised design includes six group-level observations. Please correct this. In the Methods, lines 217–219, please correct “brsm” to “brms.” In the Supporting Information, lines 663–665 and 677–679, the statement that Bulk and Tail ESS are “similar to effect sizes” is incorrect. ESS refers to effective sample size, not effect size. Please correct this in the supplementary table captions. In the Methods, lines 210–215, and Supporting Information, lines 645–649 and 656–660, the ROPE boundaries are now reported, which resolves the earlier concern. However, the main text should clarify whether the ROPE is defined on a standardized scale, consistent with the Supplementary figure captions.

Thank you for these suggestions.

We have corrected all remaining mentions of three study groups to six.

We have corrected all typos for “brms”.

We have made more clear our description of what Bulk and Tail are (Lines 688-690, 696-799, 703-706)

We have made sure that the ROPE is described consistently in the figure captions and the main text (Line 216).

Several small textual, figure, and numerical inconsistencies should also be corrected before publication.

In the Abstract, lines 25–27, the species name appears misspelled as “Phylostomus hastattus.” Please correct to Phyllostomus hastatus.

Thank you for catching this, this has been corrected.

In the Introduction, lines 44–46, please correct “individualsfrom.”

Thank you, this has been corrected.

In the Methods, lines 153–154, please correct the sentence break in “Mammalogists. and were approved…”

Thank you, we have removed the unnecessary period.

In the Methods, lines 163–166, please correct the typographical artefact “ly?the harem male.” In the Results, lines 302–307, the text refers to “H-EV4.4,” but the Methods and Table 2 list “H-EV4.2.” Please correct for consistency.

Thank you, this has been revised.

In the Figure 2 caption, lines 348–352, the caption refers to duration of allogrooming bouts in “the two harem groups,” but the revised study includes three harem groups.

Thank you, this has been revised.

In the Supporting Information, lines 631–635, the caption still refers to “three focal social groups,” although the revised manuscript discusses six group-level observations.

All captions for figures and supporting information have been updated.

Please also check numerical consistency between the Results and Table 1. In the Results, lines 225–230, the authors report 394 bachelor-group agonistic interactions, but Table 1 appears to list 386 bachelor male–bachelor male interactions. Similarly, the Results report 197 allogrooming instances, whereas Table 1 appears to list 196 female–female allogrooming interactions. If these differences are due to unknown individuals, excluded classifications, or rounding/counting decisions, this should be explained.

Thank you for noticing this, and we have rectified the inconsistencies so that the reported values in the tables and the text align.

For the supplementary allogrooming probability figure, the y-axis should be labelled more clearly as “Predicted probability of observing allogrooming in a 10-min interval.” The axis should ideally be bounded from 0 to 1, and group labels should be standardized to match the manuscript.

This figure has been remade with the axes shifted so they are strictly bound between 0 and 1, group labels modified, and the title changed as suggested.
