# Supplementary material for: Affiliative and agonistic interactions distinguish sex-biased social groups of greater spear-nosed bats, Phyllostomus hastatus
Source: PLoS One. 2026 Aug 28;21(8):e0356790. doi: 10.1371/journal.pone.0356790 (PMC13524302; doi:10.1371/journal.pone.0356790)
Supplement: Peer review history

# Peer review: Decision Letter 0

**DOI:** 10.1371/journal.pone.0356790.r001

**Authors:** Anindita Bhadra

7 Apr 2026

Dear Dr. Hex,

Thank you for submitting your manuscript to PLOS ONE. After careful consideration, we feel that it has merit but does not fully meet PLOS ONE’s publication criteria as it currently stands. Therefore, we invite you to submit a revised version of the manuscript that addresses the points raised during the review process.

Please submit your revised manuscript by May 22 2026 11:59PM. If you will need more time than this to complete your revisions, please reply to this message or contact the journal office at plosone@plos.org. When you're ready to submit your revision, log on to https://www.editorialmanager.com/pone/ and select the 'Submissions Needing Revision' folder to locate your manuscript file.

- A letter that responds to each point raised by the academic editor and reviewer(s). You should upload this letter as a separate file labeled 'Response to Reviewers'.
- A marked-up copy of your manuscript that highlights changes made to the original version. You should upload this as a separate file labeled 'Revised Manuscript with Track Changes'.
- An unmarked version of your revised paper without tracked changes. You should upload this as a separate file labeled 'Manuscript'.

If you would like to make changes to your financial disclosure, please include your updated statement in your cover letter. Guidelines for resubmitting your figure files are available below the reviewer comments at the end of this letter.

If applicable, we recommend that you deposit your laboratory protocols in protocols.io to enhance the reproducibility of your results. Protocols.io assigns your protocol its own identifier (DOI) so that it can be cited independently in the future. For instructions see: https://journals.plos.org/plosone/s/submission-guidelines#loc-laboratory-protocols. Additionally, PLOS ONE offers an option for publishing peer-reviewed Lab Protocol articles, which describe protocols hosted on protocols.io. Read more information on sharing protocols at https://plos.org/protocols?utm_medium=editorial-email&utm_source=authorletters&utm_campaign=protocols.

As the corresponding author, your ORCID iD is verified in the submission system and will appear in the published article. PLOS supports the use of ORCID, and we encourage all coauthors to register for an ORCID iD and use it as well. Please encourage your coauthors to verify their ORCID iD within the submission system before final acceptance, as unverified ORCID iDs will not appear in the published article. *Only*  the individual author can complete the verification step; PLOS staff *cannot*  verify ORCID iDs on behalf of authors.

We look forward to receiving your revised manuscript.

Kind regards,

Anindita Bhadra, PhD

Academic Editor

PLOS One

Journal Requirements:

When submitting your revision, we need you to address these additional requirements.

1. Please ensure that your manuscript meets PLOS ONE's style requirements, including those for file naming. The PLOS ONE style templates can be found at

https://journals.plos.org/plosone/s/file?id=wjVg/PLOSOne_formatting_sample_main_body.pdf and

https://journals.plos.org/plosone/s/file?id=ba62/PLOSOne_formatting_sample_title_authors_affiliations.pdf

2. In your Methods section, please provide additional information regarding the permits you obtained for the work. Please ensure you have included the full name of the authority that approved the field site access and, if no permits were required, a brief statement explaining why.

3. Thank you for stating the following in the Acknowledgments Section of your manuscript:

“This work was supported by a grant (R33-AG078474) from the National Institute of Aging.”

We note that you have provided funding information that is not currently declared in your Funding Statement. However, funding information should not appear in the Acknowledgments section or other areas of your manuscript. We will only publish funding information present in the Funding Statement section of the online submission form.

Please remove any funding-related text from the manuscript and let us know how you would like to update your Funding Statement. Currently, your Funding Statement reads as follows:

“R33-AG078474 from the National Institute of Aging”

Please include your amended statements within your cover letter; we will change the online submission form on your behalf.

4. Thank you for stating the following financial disclosure:

“R33-AG078474 from the National Institute of Aging”

Please state what role the funders took in the study.  If the funders had no role, please state: "The funders had no role in study design, data collection and analysis, decision to publish, or preparation of the manuscript."

If this statement is not correct you must amend it as needed.

Please include this amended Role of Funder statement in your cover letter; we will change the online submission form on your behalf.

5. We note that Figure 1 in your submission contain copyrighted images. All PLOS content is published under the Creative Commons Attribution License (CC BY 4.0), which means that the manuscript, images, and Supporting Information files will be freely available online, and any third party is permitted to access, download, copy, distribute, and use these materials in any way, even commercially, with proper attribution. For more information, see our copyright guidelines: http://journals.plos.org/plosone/s/licenses-and-copyright.

We require you to either (1) present written permission from the copyright holder to publish these figures specifically under the CC BY 4.0 license, or (2) remove the figures from your submission:

1. You may seek permission from the original copyright holder of Figure(s) [#] to publish the content specifically under the CC BY 4.0 license.

We recommend that you contact the original copyright holder with the Content Permission Form (http://journals.plos.org/plosone/s/file?id=7c09/content-permission-form.pdf) and the following text:

“I request permission for the open-access journal PLOS ONE to publish XXX under the Creative Commons Attribution License (CCAL) CC BY 4.0 (http://creativecommons.org/licenses/by/4.0/). Please be aware that this license allows unrestricted use and distribution, even commercially, by third parties. Please reply and provide explicit written permission to publish XXX under a CC BY license and complete the attached form.”

Please upload the completed Content Permission Form or other proof of granted permissions as an "Other" file with your submission.

In the figure caption of the copyrighted figure, please include the following text: “Reprinted from [ref] under a CC BY license, with permission from [name of publisher], original copyright [original copyright year].”

2. If you are unable to obtain permission from the original copyright holder to publish these figures under the CC BY 4.0 license or if the copyright holder’s requirements are incompatible with the CC BY 4.0 license, please either i) remove the figure or ii) supply a replacement figure that complies with the CC BY 4.0 license. Please check copyright information on all replacement figures and update the figure caption with source information. If applicable, please specify in the figure caption text when a figure is similar but not identical to the original image and is therefore for illustrative purposes only.

6. Please include captions for your Supporting Information files at the end of your manuscript, and update any in-text citations to match accordingly. Please see our Supporting Information guidelines for more information: http://journals.plos.org/plosone/s/supporting-information.

7. If the reviewer comments include a recommendation to cite specific previously published works, please review and evaluate these publications to determine whether they are relevant and should be cited. There is no requirement to cite these works unless the editor has indicated otherwise.

Additional Editor Comments:

Dear Authors,

I have now received feedback from two reviewers. While reviewer 2 has recommended minor revisions, they have pointed out to their comments to me that the sampling regime for this study is an issue, but the study can be considered as a positive first step. You will see that reviewer 1 has made several suggestions regarding the reporting of the sampling regime and statistics, which I think are good suggestions. I hope you can take these comments into consideration and revise your manuscript for further reviewing. I look forward to receiving your revised manuscript.

[Note: HTML markup is below. Please do not edit.]

Reviewers' comments:

Reviewer's Responses to Questions

**Comments to the Author**

1. Is the manuscript technically sound, and do the data support the conclusions?

Reviewer #1: Partly

Reviewer #2: Yes

**********

2. Has the statistical analysis been performed appropriately and rigorously?

Reviewer #1: No

Reviewer #2: Yes

**********

3. Have the authors made all data underlying the findings in their manuscript fully available?

The PLOS Data policy

Reviewer #1: Yes

Reviewer #2: Yes

**********

4. Is the manuscript presented in an intelligible fashion and written in standard English?

Reviewer #1: Yes

Reviewer #2: Yes

**********

Reviewer #1: I would like to thank the authors for the opportunity to review this study on the social environment and allostatic load of Phyllostomus hastatus. The integration of high-resolution 4K infrared video with cutting-edge DNA methylation age estimation represents a commendable technical effort to bridge behavioral ethology with physiological aging. The description of male "wing-locking" and the complex "allogrooming huddles" in females provide valuable new insights into the social landscape of this species.

However, the primary concern of this review is a "scaling discrepancy" between the provided data and the broad conclusions regarding chronic survival. Specifically, the study relies on an 11-day behavioral "snapshot" to support claims of lifetime allostatic load and chronic physiological wear-and-tear. Furthermore, the limited group-level replication (using only a single bachelor group) and the application of Bayesian mixed-effects models with a very low number of random effect levels (N=3) raise questions regarding the generalizability and statistical stability of the findings.

While the behavioral observations are rich and technically sound, the manuscript would benefit significantly from a more cautious framing of the causal links between short-term breeding-season aggression and long-term mortality. My detailed comments below are intended to help the authors refine these interpretations and address the statistical limitations inherent in the current study design.

Introduction

L35: The authors mention that sociality evolves partly to manage "predation dilution”. In the comparison between harem groups (in elevator shafts) and bachelors (in stairwells), is there a difference in predation risk or environmental stability? If bachelors are more exposed to predators or the elements, this could contribute to mortality independent of agonistic interactions. Please discuss if these ecological factors were considered.

L44-46: The authors state that allostatic load results from "chronic, day-to-day exposure" to social stimuli. However, the study duration is only 11 days (L116).

Please clarify how 11 days of observation can be characterized as "chronic" exposure, or revise the text to acknowledge that this study captures a acute peak in social tension (breeding season) rather than a chronic, year-round baseline.

L48-50: Since this study does not measure these physiological markers directly, please ensure the Discussion does not over-interpret "wing-locking" or "grooming" as direct proxies for these complex internal processes without citing the specific limitations of behavioral-only data.

Methodology

L119-121, L125: The study compares two harem groups against only one bachelor group. In behavioral ecology, an N=1 for a social niche makes it difficult to distinguish between a species-wide trait and the unique "culture" or personality dynamics of that specific group.

Please justify the inclusion of only one bachelor group and discuss how this limited replication affects the generalizability of the "male-as-stressful" claim.

L168-170: The authors assume all unbanded individuals in the bachelor group are male. However, the capture of a female in that exact location indicates a non-negligible presence (~7.7%) of females in supposed male-only spaces.

Please provide a sensitivity analysis or clarify the proportion of interactions involving "assumed" males. This is critical for validating the claim that the observed agonistic behaviors are strictly sex-specific.

L116- L118: The data collection period is limited to 11 days during the breeding season. The abstract, however, links these interactions to long-term "allostatic load" and "survival" (L30-31).

Please temper the discussion to acknowledge that these data represent a seasonal "snapshot." Linking 11 days of breeding-season behavior to lifetime physical wear and tear requires a more cautious interpretation.

L145-146: There is a significant difference in total footage hours: 40h for harems versus 25.5h for bachelors.

Please clarify if the observation windows (AM vs. PM) were perfectly balanced across groups. A lower sampling effort for bachelors increases the risk of "burst bias," where a single morning of high activity could disproportionately skew the results.

L166-167: Identifying the "sexually dimorphic chest gland" via infrared video in a crowded roost is technically challenging.

Please report an inter-rater reliability score (e.g., Cohen’s Kappa) to demonstrate that multiple coders reached the same conclusions regarding the sex and status of unbanded individuals.

Statistical analysis

L180, L185: The authors utilize "group ID" as a random effect (L180, L185). In Bayesian mixed-effects modeling, estimating the variance of a random effect from only three levels is mathematically unstable.

Please justify the use of a random effect with only three levels (N=3). Alternatively, consider treating "Group ID" as a fixed effect to provide more stable estimates (McElreath, 2018).

L188-189” The authors use the Region of Practical Equivalence (ROPE) to determine significance but do not define the specific boundaries used for this "null" region.

Please state the specific range used to define the ROPE so the rejection of the null hypothesis (L192) can be independently verified.

L194-195: The manuscript uses "descriptive statistics" for allogrooming because zero instances were observed in bachelors.

Please include the bachelor group in a comparative model (e.g., a Bayesian Hurdle model). This would allow for a formal statistical contrast, providing a more rigorous basis for the claim that females benefit from social buffering while males do not.

L179: The use of a zero-inflated Poisson model (L179) may not account for "overdispersion" if fights occurred in highly concentrated bursts.

Please clarify if the data was tested for overdispersion. If the variance of the aggression rate significantly exceeds the mean, a zero-inflated Negative Binomial model may be more appropriate.

Results

L250-256: Harem EV4.7 exhibited a rate of aggression more than double that of Harem CenterRight. Given this significant variation within female groups, how can the authors be certain that the higher rate in the bachelor group is a function of sex-specific niches rather than group-specific environmental factors?

L259: The text mentions Harem 'EV3.4.' Is this a typographical error intended to be Harem EV4.7 (as mentioned in L124 and L250), or was a third harem group included in the duration analysis?

L277-280: Authors describe 'allogrooming huddles' that involve grooming difficult-to-reach locations. Do you have data to suggest these huddles occur more frequently after agonistic interactions? This would provide direct evidence for the 'social buffering' hypothesis.

L265-268: Authors note that female groups have more multi-party fights (up to 5 individuals) compared to the strictly dyadic fights of males. Does this suggest that female social stress, while less physically 'intense,' is more socially 'widespread' across the group?

Discussion

L336-338: The authors provide a compelling observation regarding a spike in grooming when individuals returned covered in pollen.

Please discuss whether allogrooming in this species is primarily a functional/hygienic response to foraging residues. If the behavior is largely driven by "cleaning edible residue," it may be premature to characterize it as a "social factor contributing to differences in allostatic load" (L357) without further evidence of its psychological impact.

L354, L381: Throughout the Discussion, behavioral observations are linked to physiological wear and tear (allostatic load). While the authors acknowledge that they cannot assign causation, the concluding sentences suggest a definitive link between "day-to-day interactions" and "physiological outcomes" (L386).

Please temper the conclusion to reflect that while the correlations are consistent with the allostatic load hypothesis, this study provides no direct physiological data to confirm the mechanism in this specific population.

L356-358: The manuscript suggests that allogrooming is a key factor in buffering allostatic load and promoting survival. However, the authors also note that harem males do not participate in allogrooming (L362) yet maintain superior body condition and survival compared to bachelors (L319-320).

Please address this discrepancy. If harem males achieve high survival without allogrooming, does this imply that the absence of aggression is a more significant determinant of allostatic load than the presence of affiliative buffering?

L379: The authors cite personal observations to suggest that bachelor wounds occur year-round, justifying the 11-day "snapshot."

To strengthen this claim, please provide more detail on these observations (e.g., frequency or seasonality) or cite any available data that suggests male aggression levels do not significantly drop outside the breeding season.

Reviewer #2: Dear authors, I very much appreciate studies on social behaviour conducted in the wild. Phyllostomus hastatus is a fascinating species with respect to sociality. Although the observation period is relatively short and the sample size limited, I think your study provides valuable observations that can serve as a starting point for further research on the relationship between social environment and allostatic load (including physiological consequences and ultimately fitness). Below I provide several minor comments.

Abstract

“Valence” is only mentioned in the abstract. I recommend either removing the term or introducing it earlier in the manuscript (e.g., in the Introduction).

Methods

Line 116: “In” should be lower case.

Line 141:

Please provide the sampling rate and bit resolution of the recordings. Were recordings automatically triggered? If so, based on which threshold?

Figure S2:

Please include the spectrogram settings used (e.g., window type, FFT size, overlap). Also specify which software was used to generate the spectrograms.

Results

I have a few more general questions regarding the results:

Females appear to solicit copulations. Is this consistently the case in this species? Are there other bat species in which females actively solicit copulations? I am more familiar with species in which males either attempt forced copulations or perform elaborate courtship displays, with females making the mating decision rather than actively soliciting copulation.

Were you able to determine whether females had preferred grooming partners?

I found the fighting behaviour between males particularly interesting. The wing-lock position seems difficult for the attacked individual to counter. Are similar fighting behaviours known from other bat species?

Discussion

Line 319:

Could you briefly clarify how body condition was defined?

Line 377:

I would suggest rephrasing this statement — the observation period most likely does not fully capture seasonal variation in social behaviour.

**********

what does this mean?). If published, this will include your full peer review and any attached files.

If you choose “no”, your identity will remain anonymous but your review may still be made public.

**Do you want your identity to be public for this peer review?**  For information about this choice, including consent withdrawal, please see our Privacy Policy

Reviewer #1: No

Reviewer #2: No

**********

[NOTE: If reviewer comments were submitted as an attachment file, they will be attached to this email and accessible via the submission site. Please log into your account, locate the manuscript record, and check for the action link "View Attachments". If this link does not appear, there are no attachment files.]

To ensure your figures meet our technical requirements, please review our figure guidelines: https://journals.plos.org/plosone/s/figures

You may also use PLOS’s free figure tool, NAAS, to help you prepare publication quality figures: https://journals.plos.org/plosone/s/figures#loc-tools-for-figure-preparation.

NAAS will assess whether your figures meet our technical requirements by comparing each figure against our figure specifications.

# Author comment: Author response to Decision Letter 1

**DOI:** 10.1371/journal.pone.0356790.r002

19 May 2026

Reviewer #1:

I would like to thank the authors for the opportunity to review this study on the social environment and allostatic load of Phyllostomus hastatus. The integration of high-resolution 4K infrared video with cutting-edge DNA methylation age estimation represents a commendable technical effort to bridge behavioral ethology with physiological aging. The description of male "wing-locking" and the complex "allogrooming huddles" in females provide valuable new insights into the social landscape of this species.

However, the primary concern of this review is a "scaling discrepancy" between the provided data and the broad conclusions regarding chronic survival. Specifically, the study relies on an 11-day behavioral "snapshot" to support claims of lifetime allostatic load and chronic physiological wear-and-tear. Furthermore, the limited group-level replication (using only a single bachelor group) and the application of Bayesian mixed-effects models with a very low number of random effect levels (N=3) raise questions regarding the generalizability and statistical stability of the findings.

While the behavioral observations are rich and technically sound, the manuscript would benefit significantly from a more cautious framing of the causal links between short-term breeding-season aggression and long-term mortality. My detailed comments below are intended to help the authors refine these interpretations and address the statistical limitations inherent in the current study design.

We thank you for your helpful comments and suggestions for strengthening our analyses, as well as for refining our framing to make the scope of our study more clear. We have responded to your suggestions in greater detail below, and all changes to the manuscript are highlighted in green.

Introduction

L35: The authors mention that sociality evolves partly to manage "predation dilution”. In the comparison between harem groups (in elevator shafts) and bachelors (in stairwells), is there a difference in predation risk or environmental stability? If bachelors are more exposed to predators or the elements, this could contribute to mortality independent of agonistic interactions. Please discuss if these ecological factors were considered.

Thank you for this comment. The elevator shaft and stairwell are directly next to one another inside the building, and we do not have any evidence to suggest that one is more exposed to the elements or potential predators than the other. However, whether or not predation or other ecological factors may contribute to differences in mortality was outside of the scope of our current study, which was to characterize differences in the social environment of bachelor and harem groups.

L44-46: The authors state that allostatic load results from "chronic, day-to-day exposure" to social stimuli. However, the study duration is only 11 days (L116). Please clarify how 11 days of observation can be characterized as "chronic" exposure, or revise the text to acknowledge that this study captures a acute peak in social tension (breeding season) rather than a chronic, year-round baseline.

We would like to apologize for having not made this clear in our initial manuscript, and would like to clarify that the intent of our study was not to test hypotheses regarding allostatic load. Rather the concept of allostatic load as a potential consequence of chronic exposure to day-to-day interactions was mentioned to provide background for one reason why it is important to understand the social environment in which animals live and how this may differ between individuals as a consequence of their phenotype. To that end, this study was meant to be a first step toward understanding the types of interactions experienced by individuals in two different social environments, harem and bachelor groups, and whether they differ. Therefore, our snap-shot was not meant to represent chronic exposure, but to provide an initial characterization of differences between social environments in this species to inform future studies. In the text we have endeavored throughout the introduction and discussion to make more explicit that our discussion of allostatic load and social buffering is meant to provide context and potential bases of implications for our behavioral results.

L48-50: Since this study does not measure these physiological markers directly, please ensure the Discussion does not over-interpret "wing-locking" or "grooming" as direct proxies for these complex internal processes without citing the specific limitations of behavioral-only data.

We agree that we cannot use these behaviors as direct proxies, and we have throughout made sure to emphasize the behavioral nature of this paper.

Methodology

L119-121, L125: The study compares two harem groups against only one bachelor group. In behavioral ecology, an N=1 for a social niche makes it difficult to distinguish between a species-wide trait and the unique "culture" or personality dynamics of that specific group. Please justify the inclusion of only one bachelor group and discuss how this limited replication affects the generalizability of the "male-as-stressful" claim.

To address this concern we returned to Trinidad in January, 2026 and then recorded and subsequently scored agonistic and affiliative behaviors in two more bachelor groups and one more harem group, giving us over 110 h of data on 3 harems (containing 62 females) and 3 bachelor groups (with 86 males). We recognize that this is not a large number of groups, but it is comparable to some primate field studies. Increasing the sample size further was not possible at this time because these observations were recorded as a part of a larger study investigating the biological underpinnings of sex-biased aging in bats. As this latter study necessitates capturing individuals and collecting biological samples which disturbs the roosting group, we were limited in the number of undisturbed groups to record. As there are only approximately 20 harem groups and approximately 6 bachelor groups at this site, priority needed to be given to capturing as many bats as possible for these other studies. That being said, after adding observations from more groups, our results remain largely consistent, suggesting that there may indeed be differences in the social environments of harem groups (which are predominately females) and bachelor groups (which are predominately male).

L168-170: The authors assume all unbanded individuals in the bachelor group are male. However, the capture of a female in that exact location indicates a non-negligible presence (~7.7%) of females in supposed male-only spaces. Please provide a sensitivity analysis or clarify the proportion of interactions involving "assumed" males. This is critical for validating the claim that the observed agonistic behaviors are strictly sex-specific.

We agree that we cannot with utmost confidence assume that all individuals of the bachelor groups are males. Previous banding data and published information on this species, however, gives us greater confidence that this is a heavily male-biased space. We have captured groups of bats from the same roosting location (the stairway in the building) on 4 occasions between January 2024 and January 2026. In total, we caught 56 males and 3 females (19 males, 1 female on Jan 6, 2024; 12 males, 1 female on Jan 9, 2025; 7 males, 1 female on Jan 11, 2025; and 18 males, 0 females on Jan 9, 2026). Each of the three females showed no evidence of prior lactation and had unworn teeth, as is typical of animals in their first year prior to joining a harem group, where they remain for the rest of their lives. Consequently, the probability that an unbanded bat observed in a video from that location was a male is 56/59 = 95%.

That being said, we have endeavored to make two changes in the manuscript that we believe strengthen our arguments. First, we have replaced “sex-specific social environments” with the more accurate phrase “sex-biased social environments”, as harems are female-dominated but, by definition, include the dominant male, while bachelor groups are male-dominated but may include a small number of females for short periods, especially following natal dispersal. Second, we have attempted to make it clear that we are attempting to compare the interactions typical of the two different social environments rather than sexes, per se, though it is important to note from the natural history of this species that these two ways of looking at the question are intertwined. The few females that live in bachelor groups do so only for a short period of time before joining a harem group, while the majority of males live in bachelor groups their entire lives, and even harem males spend several years in a bachelor group. Therefore, while harem and bachelor groups are not necessarily sex-exclusive, membership in each of these group types and the amount of time spent within them is certainly strongly sex-biased and represent fundamental aspects of sex-specific social experiences.

L116- L118: The data collection period is limited to 11 days during the breeding season. The abstract, however, links these interactions to long-term "allostatic load" and "survival" (L30-31). Please temper the discussion to acknowledge that these data represent a seasonal "snapshot." Linking 11 days of breeding-season behavior to lifetime physical wear and tear requires a more cautious interpretation.

As noted above, we added a second year of observation, which spanned 10 additional days of observations. While both sets of observations were conducted during the breeding period, this period should include aggressive interactions given that this is when mating occurs.

Nevertheless, we agree that we are not able to draw direct conclusions about a lifetime of physical wear and tear. We have endeavored to make it clear that this is meant to be a first step in quantifying the behavioral side of the question and thereby motivate future studies capable of identifying causal connections. However, without first knowing whether there were any differences in the social environments experienced by males and females, there would be little justification to further investigate the role of the social environment.

L145-146: There is a significant difference in total footage hours: 40h for harems versus 25.5h for bachelors. Please clarify if the observation windows (AM vs. PM) were perfectly balanced across groups. A lower sampling effort for bachelors increases the risk of "burst bias," where a single morning of high activity could disproportionately skew the results.

Again, as noted above we have added a second year of observation, and three more focal groups (two bachelor groups and one harem group), thereby increasing our total footage hours to 52 h and 62 h for harem groups and bachelor groups, respectively.

That being said, we attempted to balance our data collection efforts across these time periods for harem and bachelor groups, though this did not result in perfectly balanced samples. We have more PM samples for harem groups than for bachelor groups. This is in part due to bachelor males remaining out of the roost longer than harem members. We only coded videos for which there was at least one individual in the roost, and while the harem groups were typically empty for about an hour between 19:00-20:00, roosts occupied by bachelor groups were often completely empty beginning at 18:00 and continued to be so even for videos taken after 20:00. Therefore, members of harem groups were simply present in the roost for more of the observation time in the evening.

Below is the distribution of number of 30 min videos in which at least one individual was in the roost for harem and bachelor groups in our sample.

AM PM

Bachelor group 30 31

Harem group 32 57

L166-167: Identifying the "sexually dimorphic chest gland" via infrared video in a crowded roost is technically challenging. Please report an inter-rater reliability score (e.g., Cohen’s Kappa) to demonstrate that multiple coders reached the same conclusions regarding the sex and status of unbanded individuals.

All but two videos were coded by a single observer (SH) and those two videos were conducted on a harem group rather than a bachelor group, so conducting inter-rater reliability such as Cohen’s Kappa is not appropriate. However, the sexually dimorphic chest gland is rather easy to identify when the individual raises their head to look down, which occurs frequently. In males, much more of the throat is bare and therefore appears brighter in the infrared-illuminated video. Below is an image taken from a harem group of a female (top left) in which the gland is just a dot, and male (top right) in which the gland is much larger and surrounded by a conspicuous white patch, which is bare skin. Further, external genitalia are readily observable because the penis is noticeable as a conspicuously bright white dot against the lower body, allowing for reliable sexing if the ventral surface is glimpsed either from the front or side (bottom right and left).

Statistical analysis

L180, L185: The authors utilize "group ID" as a random effect (L180, L185). In Bayesian mixed-effects modeling, estimating the variance of a random effect from only three levels is mathematically unstable. Please justify the use of a random effect with only three levels (N=3). Alternatively, consider treating "Group ID" as a fixed effect to provide more stable estimates (McElreath, 2018).

Thank you for this comment. As noted above, we have added data for three more social groups, which increases the number of groups for which we have observations of agonistic behavior to six. This should be sufficient to benefit from partial pooling without compromising model estimate stability.

L188-189” The authors use the Region of Practical Equivalence (ROPE) to determine significance but do not define the specific boundaries used for this "null" region. Please state the specific range used to define the ROPE so the rejection of the null hypothesis (L192) can be independently verified.

Thank you for bringing this to our attention. Our defined ROPE was included in the model outputs in the supplement, but we agree that we should have also explicitly stated it in the manuscript. We have added that our boundaries for the ROPE are -0.10 to 0.10.

L194-195: The manuscript uses "descriptive statistics" for allogrooming because zero instances were observed in bachelors. Please include the bachelor group in a comparative model (e.g., a Bayesian Hurdle model). This would allow for a formal statistical contrast, providing a more rigorous basis for the claim that females benefit from social buffering while males do not.

We appreciate this comment, and recognize that if we were trying to test specifically for social buffering or some other outcome that might be predicted by rate of allogrooming, a comparative model would be preferable. However, as mentioned above, the purpose of this manuscript is simply to describe patterns of social interaction and the differences between harem and bachelor groups. Therefore, we instead ran a brms model with Bernoulli distribution and a logit link function, converting our response variable to whether or not we observed any allogrooming (1,0) within a given observation window to produce a predicted probability of observing allogrooming in each group type. This analysis yielded the following output, which tells us there is nearly 0% chance of observing allogrooming in bachelors, and a 20-30% probability of observing it in the harem groups in a 10 min period. We have added this result to the supplement and now report the group-level median rates and durations of allogrooming.

L179: The use of a zero-inflated Poisson model (L179) may not account for "overdispersion" if fights occurred in highly concentrated bursts. Please clarify if the data was tested for overdispersion. If the variance of the aggression rate significantly exceeds the mean, a zero-inflated Negative Binomial model may be more appropriate.

Thank you for raising this. We tested for overdispersion of the rate of aggression and as you suggested, the data were overdispersed, so we re-ran the model as a ze

Attachment Submitted filename: ReviewerResponse_R1.docx

# Peer review: Decision Letter 1

**DOI:** 10.1371/journal.pone.0356790.r003

**Authors:** Anindita Bhadra

14 Jul 2026

Dear Dr. Hex,

Thank you for submitting your manuscript to PLOS ONE. After careful consideration, we feel that it has merit but does not fully meet PLOS ONE’s publication criteria as it currently stands. Therefore, we invite you to submit a revised version of the manuscript that addresses the points raised during the review process.

Please submit your revised manuscript by Aug 28 2026 11:59PM. If you will need more time than this to complete your revisions, please reply to this message or contact the journal office at plosone@plos.org. When you're ready to submit your revision, log on to https://www.editorialmanager.com/pone/ and select the 'Submissions Needing Revision' folder to locate your manuscript file.

- A letter that responds to each point raised by the academic editor and reviewer(s). You should upload this letter as a separate file labeled 'Response to Reviewers'.
- A marked-up copy of your manuscript that highlights changes made to the original version. You should upload this as a separate file labeled 'Revised Manuscript with Track Changes'.
- An unmarked version of your revised paper without tracked changes. You should upload this as a separate file labeled 'Manuscript'.

If you would like to make changes to your financial disclosure, please include your updated statement in your cover letter. Guidelines for resubmitting your figure files are available below the reviewer comments at the end of this letter.

If applicable, we recommend that you deposit your laboratory protocols in protocols.io to enhance the reproducibility of your results. Protocols.io assigns your protocol its own identifier (DOI) so that it can be cited independently in the future. For instructions see: https://journals.plos.org/plosone/s/submission-guidelines#loc-laboratory-protocols. Additionally, PLOS ONE offers an option for publishing peer-reviewed Lab Protocol articles, which describe protocols hosted on protocols.io. Read more information on sharing protocols at https://plos.org/protocols?utm_medium=editorial-email&utm_source=authorletters&utm_campaign=protocols.

As the corresponding author, your ORCID iD is verified in the submission system and will appear in the published article. PLOS supports the use of ORCID, and we encourage all coauthors to register for an ORCID iD and use it as well. Please encourage your coauthors to verify their ORCID iD within the submission system before final acceptance, as unverified ORCID iDs will not appear in the published article. *Only*  the individual author can complete the verification step; PLOS staff *cannot*  verify ORCID iDs on behalf of authors.

We look forward to receiving your revised manuscript.

Kind regards,

Anindita Bhadra, PhD

Academic Editor

PLOS One

Journal Requirements:

If the reviewer comments include a recommendation to cite specific previously published works, please review and evaluate these publications to determine whether they are relevant and should be cited. There is no requirement to cite these works unless the editor has indicated otherwise.

Please review your reference list to ensure that it is complete and correct. If you have cited papers that have been retracted, please include the rationale for doing so in the manuscript text, or remove these references and replace them with relevant current references. Any changes to the reference list should be mentioned in the rebuttal letter that accompanies your revised manuscript. If you need to cite a retracted article, indicate the article’s retracted status in the References list and also include a citation and full reference for the retraction notice.

Additional Editor Comments (if provided):

I am happy to see the revised version of the manuscript. Please respond to Reviewer 1, and submit your revised manuscript.

[Note: HTML markup is below. Please do not edit.]

Reviewers' comments:

Reviewer's Responses to Questions

**Comments to the Author**

Reviewer #1: All comments have been addressed

Reviewer #2: All comments have been addressed

**********

2. Is the manuscript technically sound, and do the data support the conclusions?

Reviewer #1: Yes

Reviewer #2: (No Response)

**********

3. Has the statistical analysis been performed appropriately and rigorously?

Reviewer #1: Yes

Reviewer #2: (No Response)

**********

4. Have the authors made all data underlying the findings in their manuscript fully available?

The PLOS Data policy

Reviewer #1: Yes

Reviewer #2: (No Response)

**********

5. Is the manuscript presented in an intelligible fashion and written in standard English?

Reviewer #1: Yes

Reviewer #2: (No Response)

**********

Reviewer #1: I thank the authors for their careful and constructive revision. The revised manuscript is substantially improved. In particular, the addition of a second observation period and additional harem and bachelor-group observations directly addresses my earlier concern about limited group-level replication. The revised dataset now provides a more convincing basis for comparing affiliative and agonistic interactions across sex-biased social environments. I also appreciate the authors’ revision of the aggression-rate model to account for overdispersion, their addition of a model for allogrooming occurrence, and their more cautious framing of the behavioural nature of the study.

Overall, I find the manuscript much stronger and closer to publication. The behavioural observations remain valuable, especially the detailed descriptions of male “wing-locking” and female allogrooming. However, I recommend minor revision before acceptance. My remaining concerns are not about the central behavioural pattern, but about ensuring that the interpretation remains appropriately cautious and that several inconsistencies introduced or retained during revision are corrected.

The authors have clarified that the study does not directly test allostatic load, social buffering, or survival mechanisms. This is a substantial improvement. However, the Abstract and final Discussion still give considerable prominence to the possibility that the observed behavioural differences contribute to allostatic load and survival differences. In the Abstract, lines 34–39, the sentence linking the behavioural differences to allostatic load and survival should be tempered slightly further. I recommend phrasing this as a hypothesis generated by the present behavioural data, or as a pattern consistent with possible future tests, rather than implying that the present study demonstrates such links. Similarly, in the Discussion, lines 448–455, the authors appropriately acknowledge that causal links cannot be drawn, but the final sentence still implies that the present study characterizes how day-to-day interactions culminate into physiological outcomes. Please revise this to state that the behavioural findings generate testable hypotheses about possible physiological consequences.

In the Methods, lines 139–141, the manuscript states that harem groups roost in the elevator shaft and bachelor groups roost in the adjacent stairwell. In the response letter, the authors further explain that they have no evidence that one location is more exposed to predators or environmental conditions than the other. This clarification should be added briefly to the manuscript, either in the Methods or Discussion, so that readers can evaluate whether roost-location differences may confound the harem–bachelor comparison.

The expanded dataset is a major improvement. However, in the Methods, lines 136–150, one bachelor roost location, B-ST3.4, was sampled in both 2025 and 2026 and treated as independent because bachelor-group membership is presumed to be labile. This may be reasonable, but it should remain explicit wherever the manuscript refers to “three bachelor groups.” The conclusions should be framed as evidence from sex-biased social environments at this study site rather than as broad species-wide generalization.

The shift from “sex-specific” to “sex-biased” social environments is appropriate. The capture data also support the claim that bachelor groups are strongly male-biased. However, in the Methods, lines 185–195, some sex assignments still rely on assumptions from roost membership and visual criteria. Please state clearly that coding was performed primarily by a single observer and that formal inter-rater reliability for sex/status assignment could not be calculated. If possible, the representative image showing the chest-gland/genitalia criteria should be included in the Supplementary Information.

In the Methods, lines 159–168, the increased total footage reduces the earlier concern about burst bias. However, because AM/PM sampling was not perfectly balanced, I recommend including the AM/PM distribution table from the response letter in the Methods or Supplement. This would make the sampling structure transparent to readers and would help show that the higher aggression rate in bachelor groups is unlikely to be an artefact of a single active time period.

In the Methods, lines 217–223, and Results, lines 338–344, the added Bernoulli model is an acceptable improvement for a descriptive study. However, the manuscript should make clear that this model estimates the probability of observing allogrooming in a 10-minute interval. It does not directly test social buffering. Any wording linking allogrooming to buffering or allostatic load should remain explicitly hypothetical.

In the Discussion, lines 416–436, the response to the harem-male discrepancy is only partly satisfactory. If harem males have better condition/survival than bachelor males but were not observed to participate in allogrooming, this suggests that reduced exposure to intense bachelor aggression may be an important alternative or complementary mechanism to allogrooming-based buffering. I recommend that the Discussion state this more clearly. Allogrooming should be framed as a potential female-specific social benefit, not as a general explanation for all harem-associated survival advantages.

In the Discussion, lines 437–448, the added capture-record information on wounds across months strengthens the argument that bachelor males may experience aggression beyond the immediate observation window. However, wound records are indirect evidence and do not quantify year-round aggression rates. The current cautious framing is mostly appropriate, but please avoid implying that equivalent aggression rates persist outside the mating season.

Several statistical and terminology issues should be corrected.

In the Methods, lines 197–200, the text still refers to “three focal groups,” although the revised design includes six group-level observations. Please correct this.

In the Methods, lines 217–219, please correct “brsm” to “brms.”

In the Supporting Information, lines 663–665 and 677–679, the statement that Bulk and Tail ESS are “similar to effect sizes” is incorrect. ESS refers to effective sample size, not effect size. Please correct this in the supplementary table captions.

In the Methods, lines 210–215, and Supporting Information, lines 645–649 and 656–660, the ROPE boundaries are now reported, which resolves the earlier concern. However, the main text should clarify whether the ROPE is defined on a standardized scale, consistent with the Supplementary figure captions.

Several small textual, figure, and numerical inconsistencies should also be corrected before publication.

In the Abstract, lines 25–27, the species name appears misspelled as “Phylostomus hastattus.” Please correct to Phyllostomus hastatus.

In the Introduction, lines 44–46, please correct “individualsfrom.”

In the Methods, lines 153–154, please correct the sentence break in “Mammalogists. and were approved…”

In the Methods, lines 163–166, please correct the typographical artefact “ly?the harem male.” In the Results, lines 302–307, the text refers to “H-EV4.4,” but the Methods and Table 2 list “H-EV4.2.” Please correct for consistency.

In the Figure 2 caption, lines 348–352, the caption refers to duration of allogrooming bouts in “the two harem groups,” but the revised study includes three harem groups.

In the Supporting Information, lines 631–635, the caption still refers to “three focal social groups,” although the revised manuscript discusses six group-level observations.

Please also check numerical consistency between the Results and Table 1. In the Results, lines 225–230, the authors report 394 bachelor-group agonistic interactions, but Table 1 appears to list 386 bachelor male–bachelor male interactions. Similarly, the Results report 197 allogrooming instances, whereas Table 1 appears to list 196 female–female allogrooming interactions. If these differences are due to unknown individuals, excluded classifications, or rounding/counting decisions, this should be explained.

For the supplementary allogrooming probability figure, the y-axis should be labelled more clearly as “Predicted probability of observing allogrooming in a 10-min interval.” The axis should ideally be bounded from 0 to 1, and group labels should be standardized to match the manuscript.

Reviewer #2: (No Response)

**********

what does this mean?). If published, this will include your full peer review and any attached files.

If you choose “no”, your identity will remain anonymous but your review may still be made public.

**Do you want your identity to be public for this peer review?**  For information about this choice, including consent withdrawal, please see our Privacy Policy

Reviewer #1: No

Reviewer #2: No

**********

[NOTE: If reviewer comments were submitted as an attachment file, they will be attached to this email and accessible via the submission site. Please log into your account, locate the manuscript record, and check for the action link "View Attachments". If this link does not appear, there are no attachment files.]

To ensure your figures meet our technical requirements, please review our figure guidelines: https://journals.plos.org/plosone/s/figures

You may also use PLOS’s free figure tool, NAAS, to help you prepare publication quality figures: https://journals.plos.org/plosone/s/figures#loc-tools-for-figure-preparation.

NAAS will assess whether your figures meet our technical requirements by comparing each figure against our figure specifications.

Attachment Submitted filename: Review report.docx

# Author comment: Author response to Decision Letter 2

**DOI:** 10.1371/journal.pone.0356790.r004

20 Jul 2026

I thank the authors for their careful and constructive revision. The revised manuscript is substantially improved. In particular, the addition of a second observation period and additional harem and bachelor-group observations directly addresses my earlier concern about limited group-level replication. The revised dataset now provides a more convincing basis for comparing affiliative and agonistic interactions across sex-biased social environments. I also appreciate the authors’ revision of the aggression-rate model to account for overdispersion, their addition of a model for allogrooming occurrence, and their more cautious framing of the behavioural nature of the study.

Overall, I find the manuscript much stronger and closer to publication. The behavioural observations remain valuable, especially the detailed descriptions of male “wing-locking” and female allogrooming. However, I recommend minor revision before acceptance. My remaining concerns are not about the central behavioural pattern, but about ensuring that the interpretation remains appropriately cautious and that several inconsistencies introduced or retained during revision are corrected.

Thank you for your encouraging comments. Below, please find our responses to your remaining suggested changes, including correcting errors introduced during revision.

Recommendation: Minor revision.

The authors have clarified that the study does not directly test allostatic load, social buffering, or survival mechanisms. This is a substantial improvement. However, the Abstract and final Discussion still give considerable prominence to the possibility that the observed behavioural differences contribute to allostatic load and survival differences. In the Abstract, lines 34–39, the sentence linking the behavioural differences to allostatic load and survival should be tempered slightly further. I recommend phrasing this as a hypothesis generated by the present behavioural data, or as a pattern consistent with possible future tests, rather than implying that the present study demonstrates such links. Similarly, in the Discussion, lines 448–455, the authors appropriately acknowledge that causal links cannot be drawn, but the final sentence still implies that the present study characterizes how day-to-day interactions culminate into physiological outcomes. Please revise this to state that the behavioural findings generate testable hypotheses about possible physiological consequences.

We have implemented the suggested wording changes to make more apparent that this paper was meant to be just one more piece in the incremental accumulation of evidence for how and why males and females differ in allostatic load and longevity in this species (Lines 33-36, Lines 458-459)

In the Methods, lines 139–141, the manuscript states that harem groups roost in the elevator shaft and bachelor groups roost in the adjacent stairwell. In the response letter, the authors further explain that they have no evidence that one location is more exposed to predators or environmental conditions than the other. This clarification should be added briefly to the manuscript, either in the Methods or Discussion, so that readers can evaluate whether roost-location differences may confound the harem–bachelor comparison.

Thank you for this suggestion, and we have added a brief clarification in lines 140-141 indicating that we have no evidence about differences in predation risk or other environmental factors.

The expanded dataset is a major improvement. However, in the Methods, lines 136–150, one bachelor roost location, B-ST3.4, was sampled in both 2025 and 2026 and treated as independent because bachelor-group membership is presumed to be labile. This may be reasonable, but it should remain explicit wherever the manuscript refers to “three bachelor groups.” The conclusions should be framed as evidence from sex-biased social environments at this study site rather than as broad species-wide generalization.

We have implemented the above suggestions, and throughout the results and conclusion removed reference to “three bachelor groups”. We have also clarified in our discussion that these patterns are only directly supported by evidence from this population (Lines 378-380, 442).

The shift from “sex-specific” to “sex-biased” social environments is appropriate. The capture data also support the claim that bachelor groups are strongly male-biased. However, in the Methods, lines 185–195, some sex assignments still rely on assumptions from roost membership and visual criteria. Please state clearly that coding was performed primarily by a single observer and that formal inter-rater reliability for sex/status assignment could not be calculated. If possible, the representative image showing the chest-gland/genitalia criteria should be included in the Supplementary Information.

We have added this information both to the main text (Lines 196-197) and have added the images showing chest glands and genitalia in the Supplemental Information as Fig S3.

In the Methods, lines 159–168, the increased total footage reduces the earlier concern about burst bias. However, because AM/PM sampling was not perfectly balanced, I recommend including the AM/PM distribution table from the response letter in the Methods or Supplement. This would make the sampling structure transparent to readers and would help show that the higher aggression rate in bachelor groups is unlikely to be an artefact of a single active time period.

Thank you for the suggestion, and we have added this table to the Supplemental materials as S1 Table.

In the Methods, lines 217–223, and Results, lines 338–344, the added Bernoulli model is an acceptable improvement for a descriptive study. However, the manuscript should make clear that this model estimates the probability of observing allogrooming in a 10-minute interval. It does not directly test social buffering. Any wording linking allogrooming to buffering or allostatic load should remain explicitly hypothetical.

We have made sure that the text and supporting material make explicit this test estimates the probability of observing allogrooming (Line 339).

In the Discussion, lines 416–436, the response to the harem-male discrepancy is only partly satisfactory. If harem males have better condition/survival than bachelor males but were not observed to participate in allogrooming, this suggests that reduced exposure to intense bachelor aggression may be an important alternative or complementary mechanism to allogrooming-based buffering. I recommend that the Discussion state this more clearly. Allogrooming should be framed as a potential female-specific social benefit, not as a general explanation for all harem-associated survival advantages.

Thank you for the suggestion. We agree that this is an important alternative hypothesis to the potential physiological benefits of living in harem groups for harem males, and we have added information about both the apparent sex-specificity of allogrooming as well as the lower exposure to aggression (Lines 427-428, 438-441).

In the Discussion, lines 437–448, the added capture-record information on wounds across months strengthens the argument that bachelor males may experience aggression beyond the immediate observation window. However, wound records are indirect evidence and do not quantify year-round aggression rates. The current cautious framing is mostly appropriate, but please avoid implying that equivalent aggression rates persist outside the mating season.

We were careful not to imply that we think aggression rates are the same throughout the year by explicitly stating that we believe our results are representative of this time of the year and reporting that instances of wounding seems to be higher during the mating season, from which our current study samples. We have attempted to use wording that does not allow further interpretation beyond what we have explicitly stated (Lines 447-453).

Several statistical and terminology issues should be corrected. In the Methods, lines 197–200, the text still refers to “three focal groups,” although the revised design includes six group-level observations. Please correct this. In the Methods, lines 217–219, please correct “brsm” to “brms.” In the Supporting Information, lines 663–665 and 677–679, the statement that Bulk and Tail ESS are “similar to effect sizes” is incorrect. ESS refers to effective sample size, not effect size. Please correct this in the supplementary table captions. In the Methods, lines 210–215, and Supporting Information, lines 645–649 and 656–660, the ROPE boundaries are now reported, which resolves the earlier concern. However, the main text should clarify whether the ROPE is defined on a standardized scale, consistent with the Supplementary figure captions.

Thank you for these suggestions.

We have corrected all remaining mentions of three study groups to six.

We have corrected all typos for “brms”.

We have made more clear our description of what Bulk and Tail are (Lines 688-690, 696-799, 703-706)

We have made sure that the ROPE is described consistently in the figure captions and the main text (Line 216).

Several small textual, figure, and numerical inconsistencies should also be corrected before publication.

In the Abstract, lines 25–27, the species name appears misspelled as “Phylostomus hastattus.” Please correct to Phyllostomus hastatus.

Thank you for catching this, this has been corrected.

In the Introduction, lines 44–46, please correct “individualsfrom.”

Thank you, this has been corrected.

In the Methods, lines 153–154, please correct the sentence break in “Mammalogists. and were approved…”

Thank you, we have removed the unnecessary period.

In the Methods, lines 163–166, please correct the typographical artefact “ly?the harem male.” In the Results, lines 302–307, the text refers to “H-EV4.4,” but the Methods and Table 2 list “H-EV4.2.” Please correct for consistency.

Thank you, this has been revised.

In the Figure 2 caption, lines 348–352, the caption refers to duration of allogrooming bouts in “the two harem groups,” but the revised study includes three harem groups.

Thank you, this has been revised.

In the Supporting Information, lines 631–635, the caption still refers to “three focal social groups,” although the revised manuscript discusses six group-level observations.

All captions for figures and supporting information have been updated.

Please also check numerical consistency between the Results and Table 1. In the Results, lines 225–230, the authors report 394 bachelor-group agonistic interactions, but Table 1 appears to list 386 bachelor male–bachelor male interactions. Similarly, the Results report 197 allogrooming instances, whereas Table 1 appears to list 196 female–female allogrooming interactions. If these differences are due to unknown individuals, excluded classifications, or rounding/counting decisions, this should be explained.

Thank you for noticing this, and we have rectified the inconsistencies so that the reported values in the tables and the text align.

For the supplementary allogrooming probability figure, the y-axis should be labelled more clearly as “Predicted probability of observing allogrooming in a 10-min interval.” The axis should ideally be bounded from 0 to 1, and group labels should be standardized to match the manuscript.

This figure has been remade with the axes shifted so they are strictly bound between 0 and 1, group labels modified, and the title changed as suggested.

Attachment Submitted filename: Reviewer Responses_R2.docx

# Peer review: Decision Letter 2

**DOI:** 10.1371/journal.pone.0356790.r005

**Authors:** Anindita Bhadra

10 Aug 2026

Affiliative and agonistic interactions distinguish sex-biased social groups of greater spear-nosed bats, Phyllostomus hastatus

PONE-D-25-60173R2

Dear Dr. Hex,

We’re pleased to inform you that your manuscript has been judged scientifically suitable for publication and will be formally accepted for publication once it meets all outstanding technical requirements.

Within one week, you’ll receive an e-mail detailing the required amendments. When these have been addressed, you’ll receive a formal acceptance letter and your manuscript will be scheduled for publication.

An invoice will be generated when your article is formally accepted. Please note, if your institution has a publishing partnership with PLOS and your article meets the relevant criteria, all or part of your publication costs will be covered. Please make sure your user information is up-to-date by logging into Editorial Manager at Editorial Manager® and clicking the ‘Update My Information' link at the top of the page. For questions related to billing, please contact billing support.

If your institution or institutions have a press office, please notify them about your upcoming paper to help maximize its impact. If they’ll be preparing press materials, please inform our press team as soon as possible -- no later than 48 hours after receiving the formal acceptance. Your manuscript will remain under strict press embargo until 2 pm Eastern Time on the date of publication. For more information, please contact onepress@plos.org.

Kind regards,

Anindita Bhadra, PhD

Academic Editor

PLOS One

Additional Editor Comments (optional):

I am happy to see that you have revised the manuscript with inputs from the reviewers. I am happy to accept the manuscript in it's current form.

Reviewers' comments:

Reviewer's Responses to Questions

**Comments to the Author**

Reviewer #1: All comments have been addressed

**********

2. Is the manuscript technically sound, and do the data support the conclusions?

Reviewer #1: Yes

**********

3. Has the statistical analysis been performed appropriately and rigorously?

Reviewer #1: Yes

**********

4. Have the authors made all data underlying the findings in their manuscript fully available?

The PLOS Data policy

Reviewer #1: Yes

**********

5. Is the manuscript presented in an intelligible fashion and written in standard English?

Reviewer #1: Yes

**********

Reviewer #1: The authors have satisfactorily addressed all the comments and concerns raised in the previous review. The revised manuscript is clear, and I have no further substantive comments. I therefore recommend acceptance of the manuscript in its present form.

**********

what does this mean?). If published, this will include your full peer review and any attached files.

If you choose “no”, your identity will remain anonymous but your review may still be made public.

**Do you want your identity to be public for this peer review?**  For information about this choice, including consent withdrawal, please see our Privacy Policy

Reviewer #1: No

**********

# Editor report: Acceptance letter

**DOI:** 10.1371/journal.pone.0356790.r006

**Authors:** Anindita Bhadra

PONE-D-25-60173R2

PLOS One

Dear Dr. Hex,

I'm pleased to inform you that your manuscript has been deemed suitable for publication in PLOS One. Congratulations! Your manuscript is now being handed over to our production team.

At this stage, our production department will prepare your paper for publication. This includes ensuring the following:

* All references, tables, and figures are properly cited

* All relevant supporting information is included in the manuscript submission,

* There are no issues that prevent the paper from being properly typeset

You will receive further instructions from the production team, including instructions on how to review your proof when it is ready. Please keep in mind that we are working through a large volume of accepted articles, so please give us a few days to review your paper and let you know the next and final steps.

Lastly, if your institution or institutions have a press office, please let them know about your upcoming paper now to help maximize its impact. If they'll be preparing press materials, please inform our press team within the next 48 hours. Your manuscript will remain under strict press embargo until 2 pm Eastern Time on the date of publication. For more information, please contact onepress@plos.org.

You will receive an invoice from PLOS for your publication fee after your manuscript has reached the completed accept phase. If you receive an email requesting payment before acceptance or for any other service, this may be a phishing scheme. Learn how to identify phishing emails and protect your accounts at https://explore.plos.org/phishing.

If we can help with anything else, please email us at customercare@plos.org.

Thank you for submitting your work to PLOS One and supporting open access.

Kind regards,

PLOS ONE Editorial Office Staff

on behalf of

Dr. Anindita Bhadra

Academic Editor

PLOS One
